# Modeling combination therapies in patient cohorts and cell cultures using correlated drug action

**DOI:** 10.1016/j.isci.2024.108905

**Published:** 2024-01-15

**Authors:** Adith S. Arun, Sung-Cheol Kim, Mehmet Eren Ahsen, Gustavo Stolovitzky

**Affiliations:** 1 Department of Applied Mathematics and Statistics, Johns Hopkins University, Baltimore, MD 21218, USA; 2 Yale School of Medicine, New Haven, CT 06510, USA; 3 Psychogenics, Inc, Paramus, NJ 07652, USA; 4 Gies College of Business, University of Illinois at Urbana-Champaign, Champaign, IL 61820, USA; 5 Carle-Illinois School of Medicine, University of Illinois at Urbana-Champaign, Champaign, IL 61820, USA; 6 DREAM Challenges, NY, NY, 10471, USA

**Keywords:** Computational chemistry, Applied computing

## Abstract

Characterizing the effect of combination therapies is vital for treating diseases like cancer. We introduce correlated drug action (CDA), a baseline model for the study of drug combinations in both cell cultures and patient populations, which assumes that the efficacy of drugs in a combination may be correlated. We apply temporal CDA (tCDA) to clinical trial data, and demonstrate the utility of this approach in identifying possible synergistic combinations and others that can be explained in terms of monotherapies. Using MCF7 cell line data, we assess combinations with dose CDA (dCDA), a model that generalizes other proposed models (e.g., Bliss response-additivity, the dose equivalence principle), and introduce Excess over CDA (EOCDA), a new metric for identifying possible synergistic combinations in cell culture.

## Highlights

• We present a method for understanding the action of combination therapies
• We model drugs combined in cells or patients as acting independently and correlatedly
• Our model provides insight into which combination therapies should be prioritized
• Deviations between experiment and model may be evidence for synergistic drug action

## Graphical abstract

**Figure:**
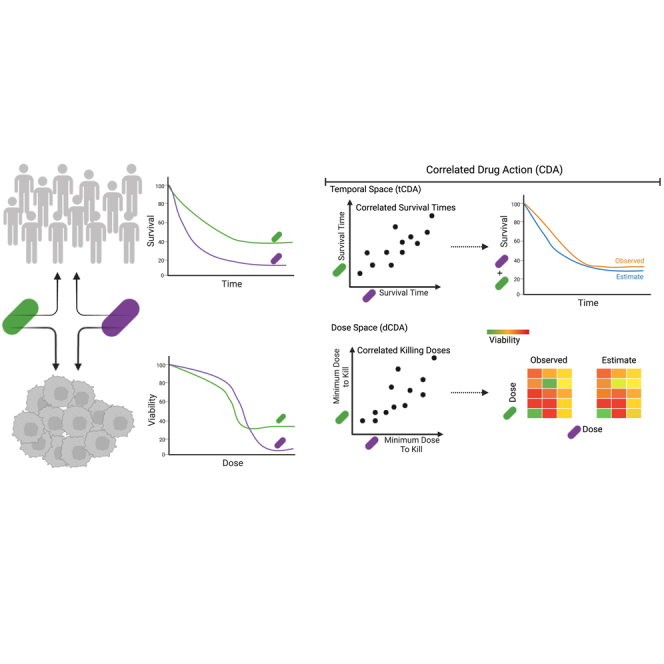

## Introduction

Combination therapy, the principle of treating a patient with multiple drugs either simultaneously or in succession, is a widely used strategy for treating conditions ranging from HIV/AIDS to cancer.^1^^,^^2^^,^^3^^,^^4^ In the case of cancer, Frei and Freirich reasoned that cancer cells could mutate and become resistant to one chemotherapy but would be less likely to become resistant to multiple different anti-cancer chemotherapy agents.^5^ Since that pioneering work, combination therapies have become an integral part of cancer treatment regimens. However, quantifying the effects of combination therapies can be experimentally inefficient given the number of possible combinations and resources such as participants, physicians, and assays needed. Thus, it is important to develop models that can characterize the effects of drugs acting in combination from the effects of the drugs acting independently. Null models, that is, models that assume the combination effect results from some sort of superposition of the individual effects of the monotherapies, can serve as a baseline for the expected effects of drugs applied in combination.^6^ For example, such models can be used as a reference to determine if a combination is more or less effective than expected.

The effect of combinations has been studied at two fundamentally different levels: *in-vitro* on cells and *in-vivo* on living organisms. Cell lines and organoids fit into the former category whereas patient-derived xenografts (PDXs) and human clinical trials are examples of the latter. At the cell culture level, research typically focuses on the dose response at a fixed time point after drug administration; at the living-organism level, research tends to focus on survival time at fixed doses. We will refer to models at the level of cultured cells as dose-space models since the primary variable to change is the dose of the monotherapies in the combination. Similarly, models describing patient populations will be referred to as temporal models given that the main endpoint is survival times.

Recently, the effects of drug combinations in patient-space have been modeled by the principle of Independent Drug Action (IDA), a model for progression-free survival that postulates that each drug in a combination acts as if the other drug wasn't present.^7^ The idea underlying IDA was first proposed by Bliss with the name of Independent Joint Action in the context of the toxicity of poisons acting in combinations.^8^ Independent action, however, does not imply that the effect of the drugs are statistically independent. As shown in [7] in the context of the IDA framework, the benefit of a combination is the highest when drug responses are uncorrelated and no benefit is expected above the better of the two drugs when the responses are perfectly correlated. Combinations of individually active therapies can increase the number of positive responses overall simply by providing several opportunities for benefit from monotherapies.^9^^,^^10^ Extending this work, a statistical derivation of a time-varying correlation formulation of IDA has been described on combination cancer therapy,^11^ we derive temporal correlated drug action (tCDA), a model based on the principles outlined by,^7^ that describes the effect of a combination as a function of individual monotherapies and a population-specific correlation coefficient. Because the models presented here will explore the role played by correlations between individual drug responses, we think that an appropriate name for this framework should be Correlated Drug Action (CDA). The ideas of CDA have previously been used to identify more robust cancer biomarkers in the face of variability in drug sensitivity in pre-clinical cancer models, showing the utility of such an approach.^12^

The tCDA approach builds on previous work in this space by presenting an analytical non-simulation-based solution which is computationally fast and easily scalable. Further, tCDA employs a non-time varying correlation coefficient which is distinct from previous work that used time-varying coefficients.^11^ The primary difference between these approaches is that tCDA is approximately valid for generic joint distribution of survival times characterized by its Spearman correlation whereas the Chen et al.'s model assumes a bivariate binomial distribution for the response of patients to either drug, characterized by a time dependent Pearson correlation. The flexibility offered with a time-varying correlation can capture some details of the joint distribtion of survival times but comes with the burden of specifying the form of this time-varying function. However, the necessary tradeoff here is that we are unable to capture time-varying effects indicating the value of both methods.

When the correlation is assumed to be zero, it implies that the two monotherapies’ response are unrelated and the tCDA model becomes the Bliss independence model.^8^^,^^13^ Expected objective response rates (ORRs) were computed from this assumption and compared to observed ORRs across 98 clinical trials testing PD-1 pathway inhibitors alone or in combination with other agents.^14^ This work demonstrated that most combination trials involving PD-1 of PR-L1 inhibitors resulted in ORRs greater than expected from PD-1/PD-L1 monotherapy alone. Looking beyond the PD-1 pathway, we investigate tCDA in settings of immunotherapy, targeted therapy, and chemotherapy.

We apply the tCDA model to public oncology clinical trial data (18 total combinations) in order to demonstrate its ability to explain the effect of clinical combination therapies and test for combinations that cannot be explained with the tCDA model. When the survival distribution of the combination can be explained by the tCDA model, the estimated correlation parameter can potentially inform us of whether they may exist sub-populations separable by a biomarker or patient covariate that can benefit more from one monotherapy over the other, or those that realize the benefit of the combination.

Human clinical trials are expensive and resource-intensive endeavors. As such, PDX experiments aim to find successful combination therapies using mice as model organisms and have been shown to predict response to therapy.^15^ Applying the IDA framework to these PDX experiments has resulted in better understanding the possible synergy of combination therapies and which combinations could work in humans.^7^ Preclinical models, specifically cell line experiments, are limited in their translational value because their results do not often correlate to clinical outcomes. However, cell lines are still often the first-place combination therapies are tested because they can help elucidate mechanism of action, are cheap and offer fast turnaround times. As such, we translate the temporal-space ideas of IDA to the dose-space of cells.

We present dose-space CDA, dCDA, as a model that describes the effect of combinations in cell cultures in terms of the dosages of each monotherapy that kill each cell in culture after a given treatment time. Like tCDA, dCDA estimates a correlation between the joint distribution of dosages required to kill cells for each monotherapy. The dCDA model in cell cultures is similar to the model for ORR in patient cohorts where both models are insensitive to the choice of joint distribution.^11^ However, tCDA and ORR models in^11^ yield a null model for responses in heterogeneous patient populations whereas dCDA are applied to isogenic cell lines with limited cellular variation. Conceptually, dCDA is an extension of Bliss response-additivity and Loewe additivity.^13^^,^^16^

Rather than introducing an entirely new model in an area that has seen much debate,^17^ the dCDA model interpolates between different individual models (Bliss response-additivity, highest single agent, the dose equivalence principle and sham combination compliance). This semi-unification arises from the derivation used to develop the dCDA model (See STAR methods). Using MCF7 breast cancer cell line experiments, we show that the dose-space CDA model is useful in assessing whether there are synergistic effects in a given combination at the level of cells. To do that, we introduce excess over CDA (EOCDA), a metric to assess possible synergy, which is similar to the excess ORR metric in^14^ with the addition that excess over CDA allows for non-zero correlations. Further, we propose an approach for identifying specific doses at which combination therapies produce outlier results suggesting the presence of locally (in dose-space) synergistic combinations.

## Results

### Temporal correlated drug action as a model to explain the effect of drug combinations in human clinical trials

**Figure 1:**
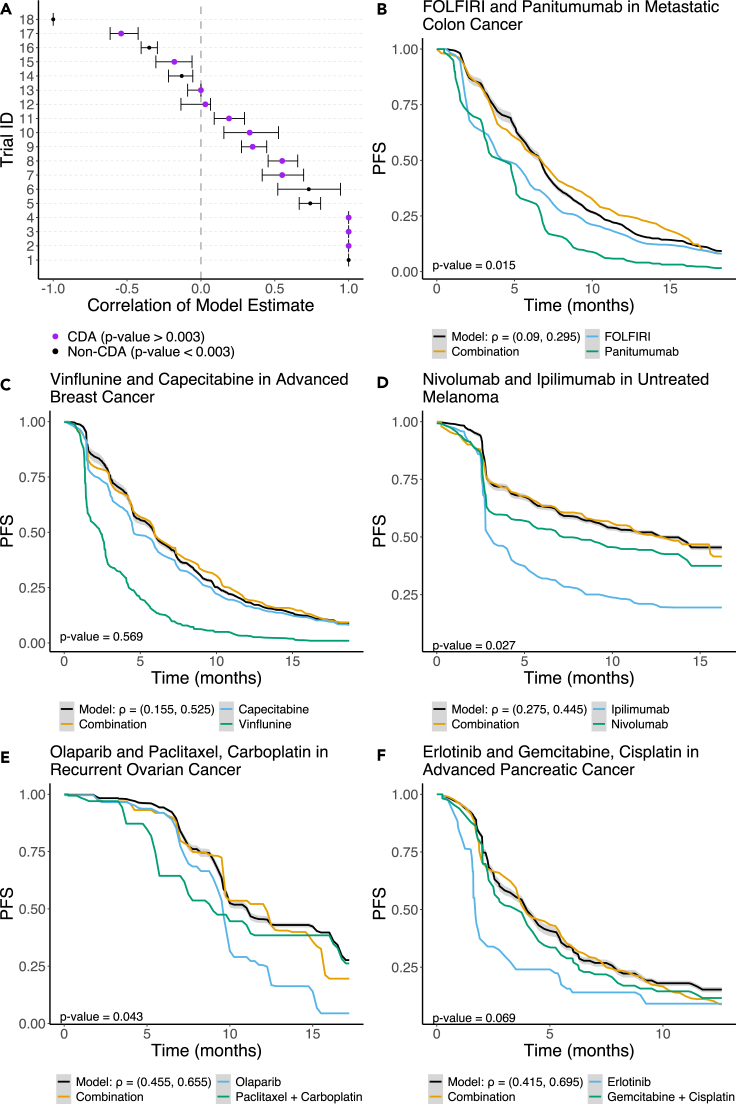
Temporal Correlated Drug Action (tCDA) model results (A) Optimal Spearman’s correlation and associated 95% confidence interval. Purple represents trials where the effect of the combination can be explained sufficiently well under the tCDA model. The trial ID’s reference the tested clinical trials, and a lookup table can be found in Table S1. (B) tCDA model estimate for the combination of FOLFIRI and Panitumumab in metastatic colon cancer. (C) tCDA model estimate for the combination of Vinflunine and Capecitabine in advanced breast cancer. (D) tCDA model estimate for the combination of Nivolumab and Ipilimumab in previously untreated melanoma. (E) tCDA model estimate for the combination of Olaparib and Paclitaxel, Carboplatin in recurrent ovarian cancer. (F) tCDA model estimate for the combination of Erlotinib and Gemcitabine, Cisplatin in advanced pancreatic cancer.

To illustrate the use of our tCDA model, we discuss its application to different combination therapy clinical trials. The simulation methods motivating the tCDA model, the closed-form expression underlying tCDA, and the statistical testing framework for assessing whether a given combination cannot be explained by the tCDA model is detailed in the STAR Methods. For the 18 different combinations tested in clinical trials and retrieved from the literature (see STAR Methods and Table S1), we estimated the optimal tCDA predicted survival curve and assessed whether the tCDA model adequately described the observed combination (see Figure 1A). We developed a procedure for estimating the correlation between single drug responses (ρ) using our closed-form mathematical expression for the tCDA model and a framework to estimate the statistical significance of the model-to-data fit (see STAR Methods). These methods are fast and scalable. In our analysis, we adjust for testing multiple hypotheses by applying a Benjamini-Hochberg correction to the individual significance level of 0.05. In 66% of the tested combinations (12 of the 18 trials), we failed to reject the tCDA model (p value > 0.003) (Figure 1A). In such cases, the estimated combination PFS curve closely tracks the experimentally observed combination (Figures 1B–1F, S3D, S4C, S4D, S5A, S6A, and S6C). We also used tCDA to analyze combinations composed of more than two drugs (e.g., Figure 1F). In 33% of the tested combinations (6 of the 18), we reject the tCDA null model (Figure 1A). These represent the combinations that perform differently than expected under the tCDA model. We observed that the tightness of the fit between the tCDA model and the actual combination can act as a red herring, distracting us from the fact that this combination is statistically significantly different than the one predicted by tCDA, as suggested by our statistical significance framework. This phenomenon can be noted in the Dabrafenib and Trametinib combination in metastatic BRAF-mutant cutaneous melanoma (Figure S6B). This encouragingly suggests a synergistic interaction which is supported by the mechanism of action of both drugs. Trametinib is a selective inhibitor of MEK1/2 activity and Dabrafenib is a potent inhibitor of BRAF and CRAF.^18^^,^^19^ Both BRAF and MEK are in the same pathway where BRAF is upstream of MEK, so drugs that inhibit these two targets provide a classical example of synergy. There are more obviously synergistic combinations, like the 5-FU and Oxaliplatin combination for advanced pancreatic cancers for which the statistical test (p value $<\;10^{-5}$) supports the visual fit and mechanistic understanding of the combination (Figure S4A).

### Dose Correlated Drug Action as a model to explain the effect of drug combinations in Cell cultures

In this section we extend the principle of IDA, originally presented to address survival of cancer patients treated with drug combinations, to the problem of determining cell viability in response to drug combinations in cell cultures and in dose space. To do so we apply the same principles that were applied for tCDA but rather than asking for the survival time of a patient under each of two drugs, we will consider the lethal dose of a cell to each of two drugs after a fixed treatment time has elapsed (typically 24, 48 or 72 h). The lethal dose, which depends on the treatment time, is a property of each cell in response to a drug. Each cell has different lethal doses for different drugs. A cell treated with a given drug at a specified dose and for a specified treatment time will be found dead (resp. alive) after that time if its lethal dose is below (resp. above) the applied dose. The extension of tCDA to cell cultures treated with a pair of drugs postulates that a cell in the culture will be viable after the treatment time has elapsed if the lethal dose of that cell for each of the monotherapies in the combination is larger than the dose of corresponding drug in the pair (see STAR Methods). Therefore, cells that had at least one of its lethal doses smaller than the applied doses in the combination will be found dead at the targeted treatment time. An important assumption in what follows is that the lethal doses of the two drugs in a combination may be correlated. The origin of this correlation could be some similarity in the mechanism of action and/or the action of some confounding variables. In cell culture, specific characteristics of a cell such as its number of mitochondria,^20^ its size, its protein content, etc., could be confounders that simultaneously affect the lethal doses of each of the two drugs. The dCDA model can be expressed as the following closed-form expression (see STAR Methods)

(Equation 1) $$\begin{array}{cc} V_{AB}\left(D_{A},D_{B},\rho \right) & =\left(1-\left|\rho \right|\right)V_{A}\left(D_{A}\right)V_{B}\left(D_{B}\right)\\ & +\left|\rho \right|\left\{ \begin{array}{ll} \min\left(V_{A}\left(D_{A}+g\left(D_{B}\right)\right),V_{B}\left(D_{B}+f\left(D_{A}\right)\right)\right) & \text{if}\;0\leq \rho \leq 1\\ \max\left(0,V_{A}\left(D_{A}\right)+V_{B}\left(D_{B}\right)-1\right) & \text{if}-1\leq \rho \leq 0 \end{array}\right. \end{array}$$

where $V_{AB}\left(D_{A},D_{B},\rho \right)$ is the dCDA predicted viability of the cell culture for the combination of drugs A and B at doses $D_{A}$ and $D_{B}$ when the Spearman’s rank correlation of the lethal doses to A and B is ρ, $V_{A}\left(D_{A}\right)$ and $V_{B}\left(D_{B}\right)$ are the dose-response curves for the cell culture treated with drugs A and B independently. The function $f\left(D_{A}\right)$ (resp. $g\left(D_{B}\right)$) is the equivalent dose of B (resp. A) that would produce the same effect in the culture viability as the dose $D_{A}$ of A (resp., the dose $D_{B}$ of B) did, and are discussed in the Supplement. For ρ=0 the dCDA model reduces to the Bliss independence model. For ρ=1 the dCDA model reduces to a modified version of the HSA model that is sham compliant which is a generalization of Loewe additivity. Indeed, when a drug is ‘combined’ with itself the lethal doses of a cell to the same drug would result in a Spearman’s correlation value of ρ=1.

**Figure 2:**
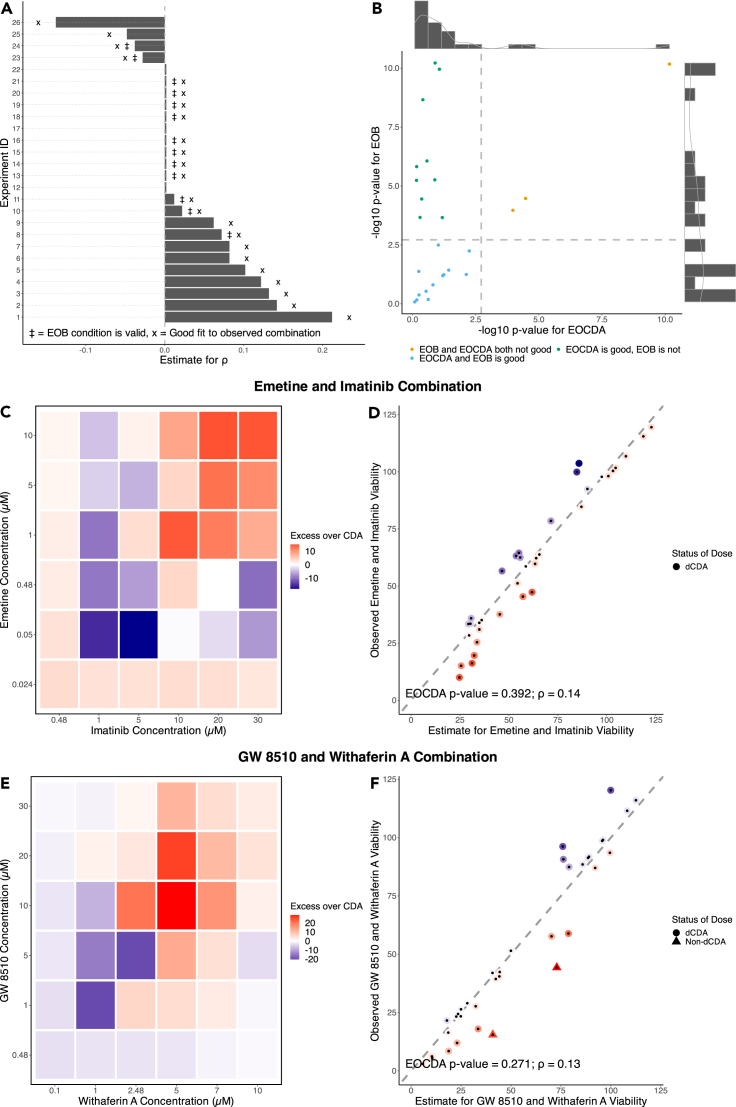
Dose-space Correlated Drug Action (dCDA) model results (A) Optimal Spearman’s correlation estimate for each *in vitro* drug combination tested on MCF7 cells under the dCDA model. Double-dagger indicates a good fit (p value > 0.01), and star indicates that Excess Over Bliss (EOB) is a valid metric (p value > 0.01). The trial ID’s correspond to a given drug combination (Table S2). (B) Joint distribution of p value statistics for Goodness of Fit and EOB conditions (Dashed lines = p value of 0.01). (C and D) Emetine and Imatinib combination results. (C) Heatmap of the EOCDA, predicted minus observed combination viabilities, metric. Positive values (more red) suggest synergy whereas negative value suggest antagonism (more blue). (D) Comparison of estimated and observed viabilities of Emetine and Imatinib in reference to the identity line (p value = 0.392). (E and F) GW 8510 and Withaferin A combination results. (E) Heatmap of EOCDA. (F) Comparison of estimated and observed viabilities (p value = 0.27). (D and F) Points are colored with the same scale as its corresponding EOCDA matrix. If the GoF p value > 0.01 for the overall combination, then each point (i.e., dose) is classified as independent or non-independent drug action.

The dCDA model requires that we find the optimal parameter ρ across a range of different doses in order to estimate the combination viability in the dCDA framework (See STAR Methods). Across the 26 experimentally tested combinations in MCF7 cells discussed below (File S2), the average Spearman’s correlation estimate is 0.03 and ranges from −0.14 to 0.21 (Figure 2A). For a given combination estimate, we assess whether the viability of a combination predicted by the dCDA model sufficiently describes the observed viability of the combination using a two-sample paired t-test (Figures 2D and 2F). We chose this method to minimize false negative rates, even when it results in some extra false positive CDA abiding combinations, for which we should have rejected the CDA null hypothesis. To compensate for this increase in false positive combinations, we will introduce later on a “local analysis” that flags specific doses of combinations as not abiding by the dCDA model.

To understand the extent to which the dCDA model captures the viability of cells in culture, we performed 26 sets of viability experiments under different doses and treatment times (see STAR Methods). 84.6% of the combinations we studied were reasonable describable under the dCDA model (Figure 2A X-labeled bars) of which a subset was also describable under the Bliss independence model (Figure 2A double-dagger-labeled bars). We apply a Benjamini-Hochberg at an individual significance level of 0.05 to adjust for multiple hypotheses being tested. The subset of cases describable by Bliss independence corresponds to the dCDA model for ρ=0. Equivalently, we can compute the difference between the estimated combination viability under the dCDA model and the observed combination viability, a metric we term the Excess over CDA (EOCDA). EOCDA is a measure of deviation from the dCDA model, and can be visualized as a heatmap where negative values (more blue) indicate an underestimation of the true viability and positive values (more red) indicate an overestimate of the true viability (Figures 2C and 2E). If a combination is describable under dCDA, then the EOCDA metric also holds as an appropriate measure (Figure 2B green and blue dots).

Classifying combinations as describable by the dCDA model or not is a global characterization and represents a measure across all of the combination viabilities at different doses taken together. We can be more granular and attempt to understand which specific doses in a dCDA compliant combination are likely synergistic or antagonistic. Let us first consider Figures 2D and 2F where each (x,y) coordinate represents the (estimate, true) combination viability and are colored according to the EOCDA color scale. Possible antagonistic drug doses in this setup will be bluer points and possible synergistic drug doses will be redder points. Through a leave-one-out cross validation re-estimation framework (See STAR Methods), we can assign each specific point (corresponding to specific doses of the two monotherapies) as locally following the dCDA model or not (p value threshold of 0.01 for minimizing rate of falsely labeling points as not following the dCDA model). For instance, in Figure 2D none of the points are locally likely synergistic or antagonistic but in Figure 2F there are two such points (both likely synergistic). Of particular note, for the cases globally describable under dCDA presented in Figures 2C and 2E and in Figures S11–S18, there are at most a few doses per combination that display locally likely synergistic or antagonistic behavior.

It is plausible, but not necessary, that the Spearman’s correlation estimate in the dCDA model describes to some extent the similarity between mechanisms of action of the individual drugs that constitute the combination. If that were the case, the larger the absolute value of the Spearman’s correlation estimate, the more similar the mechanisms of action are expected to be. Since our Spearman’s correlation estimates are on average not far from zero, we would expect that combinations describable under dCDA (GoF p value > 0.002) are composed of monotherapies whose mechanisms of action are effectively unrelated. For example, Tamoxifen targets the estrogen receptor in breast cancers whereas GW 8510 is a cyclin-dependent kinase inhibitor investigated with respect to colorectal cancer.^21^^,^^22^ These two mechanisms of action are not related in any intuitive manner. This lack of a relationship is represented by a Spearman’s correlation estimate close to zero.

The majority of the tested combinations we studied were assayed at 24 h after administering the drugs, but a select few were collected in a time-series experiment. There are two sets of combinations for which time-series viability data was collected. Experiments 21, 22, 23 correspond to Tamoxifen and Withaferin A at 12, 24, and 48 h respectively (Figure S18). The Spearman’s correlation estimate was stable over time but the EOCDA p value decreased over time (0.68, 0.15, 3.3e-5). Thus, the dose CDA model sufficiently described the combination until the 48 h time point. Experiments 5, 11, 26 correspond to Tamoxifen and Mefloquine at 12, 48, and 24 h respectively (Figure S17). The Spearman’s correlation estimates varied over time (0.12, −0.05, 0.02) but the EOCDA p values were consistently above the threshold for rejection of the dCDA null model (p value > 0.002). From both time-series experiments, we can observe that there may be undiscovered temporal dynamics at play with respect to the action of drug combinations in cell culture.

Independent of the aforementioned six experiments, we collected data for Tamoxifen and Mefloquine at 24 h in Experiment 14 and data for Tamoxifen and Withaferin A at 24 h in Experiment 12. As part of the 20 non-time series trials, we did not collect pure monotherapy viabilities but rather the viability at which the other drug in the combination was given in the lowest tested dosage. Thus, we can assess the robustness of the model to non-ideal input data by comparing Experiment 14 to Experiment 26 and Experiment 12 to Experiment 22. The Spearman’s estimate differs by 0.05 between Trial 14 and 26 and in both cases the dCDA model sufficiently describes the true data (Figure 2A). The Spearman’s estimate differs by 0.01 between Trial 6 and 25 and once again, the dCDA model p value is concordant between trials (Figure 2A). These results favorably suggest that non-ideal input data does not considerably affect the performance of the model.

Excess over Bliss (EOB), one of the most popular performance metrics for assessing the synergistic or antagonistic behavior of a combination at particular dosages, is based on Bliss independence. In order for EOB to be a valid measure, the underlying combination must be describable under Bliss independence. Given that the dCDA formula is identical to the Bliss independence formula when ρ = 0, we can formally test whether the combination can be sufficiently explained by Bliss independence (See STAR Methods). The EOB condition is valid for 50% (13 of the 26) of tested combinations (Figure 2A, double-dagger-labelled bars). Intuitively, the closer the optimal Spearman’s correlation estimate is to zero, the more likely the EOB condition is to be valid. We can also investigate the joint distribution of p value statistics for the EOB and EOCDA status of each tested combination (Figure 2B). Most of the combinations tested can be described by dCDA (blue and green dots in Figure 2B) whereas much fewer can be described by Bliss independence (blue and cyan dots in Figure 2B). For the majority of cases describable by dCDA but not Bliss (green dots), the estimated correlations are weakly positive which is sufficiently distinct from zero but likely not large enough in magnitude to reveal an obvious shared mechanism of action. At the intersection, 38.5% of combinations are both describable under dCDA and satisfy Bliss independence (Figure 2B, blue dots). It is important to note that, on the whole, the combinations describable under Bliss independence are a subset of those describable under dCDA. This highlights the strength of dCDA and EOCDA - we minimize our chances of identifying a combination as likely synergistic or antagonistic when it in fact explainable under independent but correlated joint action.

## Discussion

We introduced CDA as a baseline model to explain combinations in the temporal and dose domains. The CDA model, based on first principles, generalizes earlier models. Specifically, we discussed the tCDA model for the analysis of survival times in patient populations and the dCDA model for cell viability in cell cultures. We provided a statistical framework to assess the validity of the CDA which allows us to further classify drug combinations as likely synergistic and antagonistic, which is important to better understand combination therapies and to narrow down which combinations to investigate further. These methods may help streamline the development of novel drug combinations by assisting in ranking combinations and prioritizing which specific combinations to first test experimentally. This approach is not limited to combinations in clinical oncology. For example, this approach could be used to discover combination therapies for treating antimicrobial resistant infectious diseases.

As a model of survival times in patient populations treated with a drug combination, tCDA assumes that the monotherapies act independently but that the times that each patient would survive under each monotherapies if delivered independently are correlated.^7^^,^^8^ Our tCDA formula is based on an interpolation between full, no, and full-anticorrelation cases. This approximation yields consistent across different joint distributions of survival times (Figure S9) and therefore justify the use of the analytical form even when the correlation structure is unknown (which is the case in general). The Chen model for PFS, assumes a joint binomial distribution of drug responses,^11^ with a time dependent Pearson correlation function whereas tCDA follows from the Spearman correlation function. Despite these differences, the estimated correlations for the Nivolumab and Ipilimumab trial were between 0.2 and 0.35, all weakly positive estimates, for the tCDA, Chen, and Sorger models demonstrating the concordance between the approaches.

The analytical formula for tCDA, in comparison to simulating joint distributions as done in,^7^ offers a computational advantage in terms of speed and efficiency in fitting the model to data, which allowed us to develop a statistical procedure to test the hypothesis that the actual survival in the combination follows the tCDA model. We show that the tCDA model accurately describes a major subset of the clinically relevant combination cancer therapies remarkably well (Figures 1A and S1). The public data collected in this work to validate the tCDA model uses data from 18 clinical trials, which include nine that were described in.^7^ In order to collect clinical trial data that was in the format necessary for the tCDA model, we often had to aggregate data from up to three different clinical trials (Table S3). In these scenarios, we attempted to match cohort characteristics including sex, age, ethnicity, and previous treatment history as well as possible. Despite this effort, pooling patients across studies may lead to mismatches in demographic and clinical characteristics which could influence the likelihood of observing synergistic combinations.

We generalize the temporal-space concept of CDA by introducing the dCDA model to predict cell viability in cell cultures treated with drug combinations at different doses. We assume that for each cell in culture treated with a drug for a certain time T, there exists a lethal dose (dependent on T and varying from cell-to-cell) such that, if the drug is applied at a dose lower (resp. higher) than the lethal dose, the cell will be found alive (resp. dead) after the given treatment time T. If two drugs are applied for a time T in combination and at their respective doses, the dCDA model postulates that the only cells that survive are those for which both lethal doses are higher than the doses at which the drugs in the combination were applied. Therefore, the lethal dose for each cell in a culture in the dCDA context has the same role as the survival time for each patient in a population in the tCDA context. Like the tCDA model, the dCDA model can be formulated as a closed-form expression that is relatively insensitive to the specific form of the joint distribution. The dCDA model generalizes popular reference models proposed in the literature. The dCDA model equation equals the Bliss independence model for ρ=0 and, by leveraging the dose equivalence principle, the sham-compliant HSA for ρ=1 (See STAR Methods). This defines a spectrum of models wherein varying the Spearman’s correlation from zero to one controls whether the combination is more Bliss-like or more sham compliant HSA-like. The actual value of ρ is determined by the data, and estimated by our framework (Figure 2, and Table S2). Thus, our paradigm combines the advantages of several ways of thinking about drug interaction in the dose space.

To discover likely synergistic/antagonistic interactions between drugs, we introduced a new metric which we called Excess over Correlated Drug Action, or EOCDA, which is simply the difference between the viability estimated using the dCDA model and the observed viability. The excess ORR metric, an analogous metric to EOCDA but in temporal space, was introduced in a prior work.^11^ Positive EOCDA values correspond to likely synergistic combinations and negative EOCDA values correspond to likely antagonistic combinations (Figures 2C–2E). We compared the EOCDA metric to the often-used Excess over Bliss (EOB) metric. For both the EOCDA and EOB metrics we defined a statistical significance test to determine if the null hypothesis (EOCDA = 0 or EOB = 0) should be rejected. Therefore, for each of the tested combinations, we have a p value for the null hypothesis EOB = 0 and EOCDA = 0. Figure 2B illustrates the degree of relatedness between the two metrics. We can notice that there are more cases for which we cannot reject the null hypothesis of IDA (at a significance level of 0.01) under the dCDA when the real viability is compared to the viability estimated using Bliss independence (more blue and green dots than blue and yellow dots in Figure 2B). In other words, EOCDA reduces the chance of calling a combination likely synergistic or antagonistic compared to EOB—an important criterion when considering the experimental resources and effort that it takes to validate effective drug combinations. One limitation of EOCDA, however, is that we need the entire dose matrix with corresponding viabilities to estimate the correlation coefficient whereas EOB can be computed at individual dosages. Interestingly, for combinations describable under dCDA, we observe that there are only a few doses that are likely “locally” synergistic or antagonistic combinations in a globally independent drug combination. This plausibly identifies dosages of particular interest for further investigation in these model systems.

Tumor cells in culture, unlike tumors in patients, are relatively homogeneous since they are free of the tumor microenvironment and external milieu. Therefore, the magnitude of the dCDA estimated correlation may inform whether two drugs have similar mechanisms of action. Large magnitudes imply a strong similarity between two drugs’ mechanisms of action. In our data, all of the correlation estimates are weak implying that, from the perspective of dCDA, the drugs probably act using alternative pathways (Figure 2A). For example, GW 8510 is a cyclin-dependent kinase inhibitor whereas Withaferin A inhibits vimentin, an intermediate filament. These two mechanisms are not linked by any known direct relationship, and this supports the low Spearman’s correlation estimate given by the dCDA model (Figures 2E and 2F). More work is needed to validate the notion that similarity in the mechanism of action of two drugs can be reflected in the value of the correlation parameter as there is known variability in drug sensitivities in cell lines and could possibly be linked to confounding factors such as the size of the cells or number of mitochondria.^12^^,^^20^

There are many possible avenues for extending this work. One such extension could be the creation of a network of therapies for a given disease. Each node is a particular monotherapy and the edge width between two nodes corresponds to Spearman’s correlation estimate. This could be done for cell cultures or patient populations. From this setup, we can attempt to learn on the graph and infer the edge strength between other pairs of nodes. This could be informative in suggesting new candidates for cell line experiments or clinical trials. Additionally, we can investigate the relationship between the dose-space and temporal CDA models by testing the same cancer combination therapy in mice PDX models and patient cancer cells cultured in the lab. Finally, a more general theory that unifies both dose models and temporal models into one single model seems plausible and potentially useful.

### Limitations of the study

Our work assumes that drugs in a combination act independently, but in a correlated fashion. The correlation between the outcomes of a drug pair (survival times in tCDA and killing dose in dCDA) can be inferred indirectly by fitting the model to experiments. However, this correlation is not directly measurable, as it is a parameter that results from a theoretical construct that is not feasible in reality. For example, estimating the correlation in dCDA requires recording the dose that kills a given cell if treated with one drug and then rewind the clock and record the dose that would kill the same cell if treated with the other drug, and repeat for each cell in a culture. Further, the formulas that estimate the outcome of a combination (survival times in tCDA and dose response in dCDA) as a function of the outcomes of individual drugs are rigorously exact only under certain types of joint distributions of outcomes, but they are only approximations (albeit good ones) under other types of joint distributions. Caution should be exercised when using of the formulas presented in the main text when the correlation parameter is not close to 0 or 1. As shown in the paper, independent action with correlated outcomes describes a large fraction of drug combinations, but fails at describing the results of a few combinations, and in that sense its practicality is limited as we do not know *a priori* which combinations will be well described by CDA. Furthermore, even if we knew that the CDA model will be a good description of a combination, we would not know *a priori* which correlation parameter to use without fitting the model to the experiment. Notwithstanding the above limitation, CDA can be used to rank all combinations in a panel under the most favorable correlation and choose the most promising combinations (from a CDA perspective) for further experimentation. To test the ability of tCDA to explain the action of a combination, we made use of published clinical trial data. However, the progression-free survival curves of some trials have a discrete temporal resolution given the limited number of patients in the trial. For some combinations, we aggregated data across different clinical trials matching demographics parameters such as age, sex, and ethnicity. This aggregation may have created biases in the results for which we have not accounted.

## STAR★Methods

### Key resources table

**Table:** 

REAGENT or RESOURCE	SOURCE	IDENTIFIER
**Chemicals, peptides, and recombinant proteins**		

Eagle's Minimum Essential Medium	ATCC	Cat#30–2003
Human Recombinant Insulin	Sigma-Aldrich	Cat#11061-68-0
Fetal Bovine Serum	ATCC	Cat#30-2021
Atovaquone	Andrea Califano Lab	
Emetine	Andrea Califano Lab	
GW 8510	Andrea Califano Lab	
Imatinib	Andrea Califano Lab	
Mefloquine	Andrea Califano Lab	
MG 132	Andrea Califano Lab	
Mitoxanthrone	Andrea Califano Lab	
Nicardipine	Andrea Califano Lab	
Sanguinarine	Andrea Califano Lab	
Tamoxifen	Andrea Califano Lab	
Terfenadine	Andrea Califano Lab	
Tryphostin	Andrea Califano Lab	
AG 825	Andrea Califano Lab	
Withaferin A	Andrea Califano Lab	
Cell-Titer-Glo	Promega Corporation	Cat#G7570

**Critical commercial assays**		

MycoAlert	Lonza	Cat#LT07-701

**Deposited data**		

Generated Data	Zenodo	Accession Number: 8332696

**Experimental models: Cell lines**		

MCF-7 Cell Line	ATCC	Cat#ATCC HTB-22

**Software and algorithms**		

Perkin Elmer Envision 2104 Enhanced Luminescence Protocl	Perkin Elmer	
Code authors created to analyse data	Zenodo	Accession Number: 8332696
Web Plot Digitizer	Automeris	Version 4.6

### Resource availability

#### Lead contact

Further information and requests for resources and reagents should be directed to and will be fulfilled by the lead contact, Gustavo Stolovitzky (gustavo.stolo@gmail.com).

#### Materials availability

This study did not generate new unique reagents.

#### Data and code availability

• All the data can be found here https://github.com/aditharun/correlated-drug-action/tree/main/raw-data which is publicly accessible.
• All original code has been deposited at Zenodo and is publicly available at https://zenodo.org/records/8332697.
• Any additional information required to reanalyze the data reported in this paper is available from the lead contact upon request.

### Experimental model and study participant details

MCF7 (ATCC HTB-22) cells were obtained from ATCC. Cells were cultured according to manufacturer’s recommendations in ATCC-formulated Eagle’s Minimum Essential Medium (Catalog No. 30–2003) with 10% heat-inactivated fetal bovine serum, and 0.01 mg/ml human recombinant insulin. Growth media was changed every 3–4 days. After reaching confluence, cells were split at a ratio 1:6. Cultures were tested for mycoplasma periodically using MycoAlert (Lonza, Cat No. LT07-701) per manufacturer’s instructions. To split, media was removed, cells were washed with PBS, and trypsin-EDTA mix was added for 5 min. After detachment, cells were washed with growth media, collected into 50 ml vial, spin down at 1000 RPM, suspended in fresh media and plated into 75 cm flasks. The cells were plated at 10,000 cells per well in a clear bottom black 96-well plate (Greiner Cat. No. 655090) and a white 96-well plate (Greiner Cat. No. 655083) then they were placed in an incubator. After 24 hr, the plates were removed from the incubator and treated with drugs using the HP D300 Digital Dispenser.

### Method details

#### Collection of clinical trial data

We collected data from clinical trials using the clinicaltrials.gov query tool and scraped Progression Free Survival (PFS) data from their associated results and papers. We converted graphs into tables of values through a robust online digitizer (https://apps.automeris.io/wpd/). Though it would be best to have the original data, these files were not available to us. For a given combination, we aimed to find a single trial that tested the individual therapies and the combination. However, we also had to pull data across different trials to construct the necessary survival data. In these cases, we tried to maximize overlap in patient characteristics, dosages, and time schedules. We were able to match dosages and time schedules in the overwhelming majority of cases. We assembled data for 18 distinct combinations spanning 10 different cancer types. A detailed guide to the raw data can be found in Table S3. The tables of the raw PFS curves from the digitized plots that we used for our analysis can be found in File S1.

#### Drug combination experiments in the MCF7 cell line

##### Choice of drug combinations, dose matrix and treatment times

We applied the dCDA model to fit the viability of MCF7 cells in response to 26 drug combinations. These 26 drug combinations comprised 20 unique combinations, and 6 repeat combinations as discussed below. The 20 combinations, which included 13 unique drugs (Atovaquone, Emetine, GW 8510, Imatinib, Mefloquine, MG 132, Mitoxanthrone, Nicardipine, Sanguinarine, Tamoxifen, Terfenadine, Tyrphostin AG 825 and Withaferin A), were selected from a a pre-existing drug combination screen collected at Columbia University (Andrea Califano lab) in the context of the Library of Integrated Network-Based Cellular Signatures (LINCS) Program. This screen tested all 990 combinations of 99 drugs against 10 drugs, each combination assessed in a dose response matrix of 4x4 doses. Of these combinations we chose a subset of combinations that exhibited antagonistic, independent and synergistic behaviors, resulting in the 20 combinations reported here. The 20 drug combinations were administered at doses shown in the 6x6 dose matrices shown in Figures 2 and S10–S15, and the viability was measured at 24 hours. These 20 experiments, which we refer to as the 24h viability experiments, are reported for the first time in this paper and provided as File S2. Amongst the 20 drug pairs, two specific combinations (Tamoxifen/Withaferin A and Tamoxifen/Mefloquine) were measured at more detailed dose matrices and treatment times as reported in an earlier publication.^23^ For these two pairs, viability was measured at the doses corresponding to the 10x10 dose matrix shown in Figures S16 and S17, and at treatment times of 12h, 24h and 48h. We also measured the viability of the individual monotherapies (Tamoxifen and Mefloquine) at the 10 doses reported in the figures, at 12h, 24h, and 48h. These 6 experiments, which we will refer to as the time course viability experiments, had been previously made publicly available in;^23^ we also provide these data for convenience in File A2. All drug combination viability experiments (the 24h and time course experiments) were done in triplicate and the results for each combination were averaged. All monotherapy experiments (for the time course data) were done in duplicate and the results averaged. The dCDA model requires that we have viability for the monotherapies. We had that data for the time course data, but we lacked the equivalent data for the 24h viability experiments. Therefore to apply the dCDA model, we approximated the cell viability under individual drugs by considering the case when the other drug in the combination was measured at its lowest concentration.

#### Cell viability

Cells were treated with the appropriate drug combinations at specific dose matrices and treatment times. After the targeted drug treatment time, 100 μL of Cell-Titer-Glo (Promega Corp.) was added to the wells in the white 96-well plate and shaken at 500 rpm for 5 min. The plate was then read by the Perkin Elmer Envision 2104 using an enhanced luminescence protocol to count the number of raw luminescent units per well. For each viability experiment, the results were normalized by the luminescence measured in untreated cell cultures. The ratio of luminescence in treated vs untreated cells is what we report as cell viability.

#### tCDA model

##### Mathematical details

Temporal Correlated Drug Action (tCDA) is an independent joint action model for the action of drug combination on patients when studying their survival times. Let us assume that the effect of a therapy on a patient population can be evaluated using the distribution of survival times such as Progression Free Survival (PFS) or Overall Survival (OS). tCDA postulates that the effect of two therapies in combination is the same as the effect of the single best therapy if they had been given independently. In other words, if we knew *a priori* which of two therapies yields better results for a given patient (something that precision medicine can not predict today), tCDA postulates that the effect of administering the best monotherapy for that patient would be the same as the effect of administering the combination.

The survival times may vary from patient to patient due to different individual conditions such as tumor composition, immune system status and co-morbidities among others reasons. tCDA assumes no direct interaction between the two therapies administered to a patient. However, the hypothetical survival times on the same patient due to each of two drugs could be correlated due to the fact that the individual patient could act as a confounding factor through either the nature of the tumor, the age, the health status, etc. For example, a patient with an aggressive tumor would have a shorter survival if treated with either monotherapy, while a patient with a less aggressive tumor would survive longer with either monotherapy. In this case, the survival times under each monotherapy will be correlated.

The tCDA framework provides a baseline hypothesis for independent action of the drugs that can be used as a null hypothesis in a formal hypothesis test to classify clinical combinations as consistent with tCDA or inconsistent with tCDA. Non-tCDA combinations that are of particular interest are antagonistic or synergistic combinations.

##### Notation

For a patient in a cohort, we will denote by $t_{A}$, $t_{B}$ and $t_{AB}$ the times that the patient would survive if treated with drug A, drug B or the combination of A and B respectively. Under the tCDA model, the survival time $t_{AB}=\max\left(t_{A},t_{B}\right)$ for a given patient. Note that in practice we can only measure one of these survival times as a patient will only be treated with either treatment, and their survival will only be registered under that treatment. However, we can computationally simulate the survival times under the other treatment for the purposes of developing the tCDA model.

The survival probability that a generic patient survives a time t under treatments A, B and combination AB will be denoted by $S_{A}\left(t\right)$, $S_{B}\left(t\right)$ and $S_{AB}\left(t\right)$, and can be formally expressed as

(Equation 2) $$S_{A}\left(t\right)=\text{Prob}\left(t_{A}>t\right)$$

(Equation 3) $$S_{B}\left(t\right)=\text{Prob}\left(t_{B}>t\right)$$

(Equation 4) $$S_{AB}\left(t\right)=\text{Prob}\left(t_{AB}>t\right)$$

(Equation 5) $$t_{AB}=\text{max}(t_{A},t_{B})$$

In practice the probabilities are computed as fractions in a cohort. For example, the $S_{A}\left(t\right)$ is the fraction of patients in a cohort whose survival time was longer than t.

The hypothetical survival times $t_{A}$ and $t_{B}$ that a patient would survive under drugs A and B will in general be correlated through confounding factors. We will consider two types of correlations between these times. The Pearson correlation will be denoted by $\rho_{P}$ and the Spearman’s correlation, the correlation between the ranks, will be denoted by $\rho_{s}$.

##### Formulation of the temporal correlated drug action model

In this section we want to derive expressions for the survival probability that a patient has a survival time equal to or longer than t under treatment with both drugs A and B in terms of the survival probabilities under the monotherapies. Under the tCDA model, if a patient survives more than time t under the AB combination, we have either $t_{A}>t$ or $t_{B}>t$. Therefore, from Equation 4

(Equation 6) $$S_{AB}\left(t\right)=\text{Prob}\left(t_{A}>t\;\text{OR}\;t_{B}>t\right)$$

(Equation 7) $$\begin{array}{cc} & =1-\text{Prob}\left(t_{A}\leq t\;\text{AND}\;t_{B}\leq t\right)\text{,} \end{array}$$

where we used that the complement of a union (“OR” logical operation) of two sets is the intersection (“AND” logical operation) of the complement of the two sets. To proceed we consider separately the correlated and the uncorrelated cases.

##### Uncorrelated case

We will consider the case where the two drugs A and B are uncorrelated because they are statistically independent from one another. (In the general case, the drugs could be uncorrelated but statistically dependent). In this case, $\text{Prob}\left(t_{A}\leq t\;\text{AND}\;t_{B}\leq t\right)=\text{Prob}\left(t_{A}\leq t\right)\text{Prob}\left(t_{B}\leq t\right)$, which leads to

(Equation 8) $$S_{AB}\left(t,\rho_{s}=0\right)=S_{A}\left(t\right)+S_{B}\left(t\right)-S_{A}\left(t\right)S_{B}\left(t\right)\text{,}$$

where we used that $\text{Prob}\left(t_{A}\leq t\right)=1-S_{A}\left(t\right)$. Note that in this case, the survival under the two drugs is greater than the survival under either of the single therapies. Note the similarity between this formula and the equivalent formula for Bliss independence when computing the inhibition of a cell culture measured at a particular time after application of a drug combination. In that case the inhibition of the cell culture under the Bliss independence model is estimated as $I_{AB}=I_{A}+I_{B}-I_{A}I_{B}$.

##### Positively correlated case

Let us consider the extreme case in which for any given patient the survival time $t_{A}$ completely determines the survival time $t_{B}$, in such a way that the Spearman’s correlation between survival times under A and B treatments of all patients is 1. That means that the order of survival times of patients under treatment A is the same as the order under treatment B. Take now a patient with survival times $t_{A}$ and $t_{B}$. The fraction $S_{A}\left(t_{A}\right)$ of patients that survived more than the patient under A, should be the same as the fraction $S_{B}\left(t_{B}\right)$ of patients that survived more than the patient under B. Therefore when the Spearman’s correlation between survival times is $\rho_{s}=1$, we have that

(Equation 9) $$t_{B}=S_{B}^{-1}\left(S_{A}\left(t_{A}\right)\right)\text{.}$$

In this extreme case we can compute the survival time under the combination AB. First we note that we can express $\text{Prob}\left(t_{A}\leq t\;\text{AND}\;t_{B}\leq t\right)$ in terms of conditional probabilities as

(Equation 10) $$\text{Prob}\left(t_{A}\leq t\;\text{AND}\;t_{B}\leq t\right)=\text{Prob}\left(t_{A}\leq t|t_{B}\leq t\right)\text{Prob}\left(t_{B}\leq t\right)$$

(Equation 11) $$\begin{array}{cc} & =\text{Prob}\left(t_{B}\leq t|t_{A}\leq t\right)\text{Prob}\left(t_{A}\leq t\right)\text{.} \end{array}$$

Suppose now that $S_{B}\left(t\right)>S_{A}\left(t\right)$. This means that at time t, drug B is better than A, in that there are more patients that survived with drug B more than time t than with drug A. Because the order of survival of the patients is the same under both drugs then all the patients that did not survive under B, couldn’t have survived under A. Therefore if B is better than $A,\;\text{Prob}\left(t_{A}\leq t|t_{B}\leq t\right)=1$. Symmetrically, if A is better than $B,\;\text{Prob}\left(t_{B}\leq t|t_{A}\leq t\right)=1$.

On the other hand, when $t_{A}$ and $t_{B}$ and uncorrelated and independent, we have that $\text{Prob}\left(t_{A}\leq t|t_{B}\leq t\right)=\text{Prob}\left(t_{A}\leq t\right)$ and symmetrically, $\text{Prob}\left(t_{B}\leq t|t_{A}\leq t\right)=\text{Prob}\left(t_{B}\leq t\right)$. In the intermediate case between in which we have some correlation $\rho_{s}$, we will interpolate the conditional probabilities in such a way that the completely correlated and uncorrelated case case match the extreme cases as follows

(Equation 12) $$\text{Prob}\left(t_{A}\leq t|t_{B}\leq t\right)=\alpha \left(\rho_{s}\right)+\left(1-\alpha \left(\rho_{s}\right)\right)\text{Prob}\left(t_{A}\leq t\right)\;\text{if}\;S_{B}\left(t\right)>S_{A}\left(t\right)$$

(Equation 13) $$\text{Prob}\left(t_{B}\leq t|t_{A}\leq t\right)=\alpha \left(\rho_{s}\right)+\left(1-\alpha \left(\rho_{s}\right)\right)\text{Prob}\left(t_{B}\leq t\right)\;\text{if}\;S_{B}\left(t\right)\leq S_{A}\left(t\right)$$

where $\alpha \left(\rho_{s}=0\right)=0$, $\alpha \left(\rho_{s}=1\right)=1$ and the specific functional form in the range $0\leq \rho_{s}\leq 1$ will depend on the joint distribution of survival times.

Replacing these results in Equation 11 and using Equation 4 we find the survival under the combination AB in the case of $0\leq \rho_{s}\leq 1$ is

(Equation 14) $$S_{AB}\left(t,0\leq \rho_{s}\leq 1\right)=\left\{ \begin{array}{cc} S_{A}\left(t\right)+S_{B}\left(t\right)\left(1-S_{A}\left(t\right)\right)\left(1-\alpha \left(\rho_{s}\right)\right) & \text{if}\;S_{A}\left(t\right)>S_{B}\left(t\right)\\ S_{B}\left(t\right)+S_{A}\left(t\right)\left(1-S_{B}\left(t\right)\right)\left(1-\alpha \left(\rho_{s}\right)\right) & \text{if}\;S_{A}\left(t\right)\leq S_{B}\left(t\right) \end{array}\right.$$

##### Negatively correlated case

Let us consider the other extreme case in which for any given patient the survival t ime $t_{A}$ completely determines the survival time $t_{B}$, in such a way that the Spearman correlation, $\rho_{s}$, between survival times under A and B treatments of all patients is −1. That means that the order of survival times of patients under treatment A is the opposite as the order under treatment B. Take now a patient with survival times $t_{A}$ and $t_{B}$. Therefore, $S_{A}\left(t_{A}\right)$ represents the fraction of patients that survived more than the patient in question under treatment A; this should be the same as $1-S_{B}\left(t_{B}\right)$, the fraction of patients that died earlier than the patient in question under B. Thus, when the Spearman correlation between survival times is $\rho_{s}=-1$, we have that

(Equation 15) $$t_{B}=S_{B}^{-1}\left(1-S_{A}\left(t_{A}\right)\right)\text{.}$$

In this extreme case we can compute the survival time under the combination AB. First we note that there is a time $t^{*}$ such that all the patients that lived more than $t^{*}$ under A lived less than $t^{*}$ under B. This condition is given by $S_{A}\left(t^{*}\right)=1-S_{B}\left(t^{*}\right)$. Figure S1 shows that for $t>t^{*}$, the fraction of patients that survived more than t under A or B is the sum of those who survived more than t under A and those who survived more than t under B, given that the anticorrelation makes these groups non-overlapping. Figure S1 also shows that for t<t∗, all patients survived more than t thanks to either monotherapy or both monotherapies. Therefore for $\rho_{s}=-1$, we have that

(Equation 16) $$S_{AB}\left(t,\rho_{s}=-1\right)=\left\{ \begin{array}{ll} 1 & \text{if}\;t\leq t^{*}\\ S_{A}\left(t\right)+S_{B}\left(t\right) & \text{if}\;t>t^{*} \end{array}\right.$$

where $t^{*}$ is defined as the time that makes $S_{A}\left(t^{*}\right)+S_{B}\left(t^{*}\right)=1$.

In the range of negative correlations, we can interpolate between the cases of $\rho_{s}=0$ and $\rho_{s}=-1$, which yields

(Equation 17) $$S_{AB}\left(t,-1\leq \rho_{s}\leq 0\right)=\left\{ \begin{array}{ll} \left[S_{A}\left(t\right)+S_{B}\left(t\right)-S_{A}\left(t\right)S_{B}\left(t\right)\right]\left[1-\alpha \left(\rho_{s}\right)\right]+\alpha \left(\rho_{s}\right) & \text{if}\;t\leq t^{*}\\ S_{A}\left(t\right)+S_{B}\left(t\right)-S_{A}\left(t\right)S_{B}\left(t\right)\left[1-\alpha \left(\rho_{s}\right)\right] & \text{if}\;t>t^{*} \end{array}\right.$$

where $\alpha \left(\rho_{s}=0\right)=0$, $\alpha \left(\rho_{s}=-1\right)=1$, and the specific dependence of $\alpha \left(\rho_{s}\right)$ on $\rho_{s}$ depends in the joint distributions of times $t_{A}$ and $t_{B}$.

##### Integrated expression for the survival probability under the tCDA model

The formulas of the previous subsection can be integrated in one simple formula. Call $S_{\max}\left(t\right)=\max\left(S_{A}\left(t\right),S_{B}\left(t\right)\right)$, and $S_{\min}\left(t\right)=\min\left(S_{A}\left(t\right),S_{B}\left(t\right)\right)$. The expression for the survival probability under the tCDA model can be written as:

(Equation 18) $$S_{AB}\left(t,\rho_{s}\right)==\left\{ \begin{array}{ll} S_{\max}\left(t\right)+S_{\min}\left(t\right)\left(1-S\;\max\left(t\right)\right)\left(1-\alpha \left(\rho_{s}\right)\right) & \text{if}\;0\leq \rho_{s}\leq 1\\ \left[S_{A}\left(t\right)+S_{B}\left(t\right)-S_{A}\left(t\right)S_{B}\left(t\right)\right]\left[1-\alpha \left(\rho_{s}\right)\right]+\min\left(1,S_{A}\left(t\right)+S_{B}\left(t\right)\right)\alpha \left(\rho_{s}\right) & \text{if}\;-1\leq \rho_{s}\leq 0 \end{array}\right.$$

The model is not complete until we specify the function $\alpha \left(\rho_{s}\right)$, which is unknown except in the border cases $\rho_{s}=$-1, 0, 1 for which $\alpha \left(\rho_{s}\right)$ = is 1, 0, 1 respectively. As an approximation, we can assume that $\alpha \left(\rho_{s}\right)=|\rho_{s}|$. In the next section we will show that this is indeed the correct function for a particular joint distribution for $t_{A}$ and $t_{B}$.

##### There is a joint survival times distribution for which $\alpha \left(\rho_{s}\right)=\left|\rho_{s}\right|$

There are many possible joint distributions of $t_{A}$ and $t_{B}$ that yield the same Spearman correlation, and for each such distribution there may be a different function $\alpha \left(\rho_{s}\right)$. Therefore from the perspective of defining uniquely the tCDA model, it is necessary to specify the joint distribution of survival times. In this section we will choose such distribution and show that for it, $\alpha \left(\rho_{s}\right)=|\rho_{s}|$, which we will call the tCDA joint survival times probability density, or tCDA distribution for short.

This distribution can be thought as a mixture distribution where a fraction 0≤α≤1 of ($t_{A}$, $t_{B}$) pairs are drawn from the line $t_{B}=S_{B}^{-1}\left(S_{A}\left(t_{A}\right)\right)$ for $0\leq \rho_{s}\leq 1$ or the line $t_{B}=S_{B}^{-1}\left(1-S_{A}\left(t_{A}\right)\right)$ for $0\leq \rho_{s}\leq 1$, and the other 1−α fraction are independently drawn from the distributions $P_{A}\left(t_{A}\right)$ and $P_{B}\left(t_{B}\right)$. Formally this can be written as

(Equation 19) $$\begin{array}{cc} P\left(t_{A},t_{B}\right) & =\left(1-\alpha \right)P_{A}\left(t_{A}\right)P_{B}\left(t_{B}\right)\\ & +\alpha \left\{ \begin{array}{ll} \delta \left(S_{A}\left(t_{A}\right)-S_{B}\left(t_{B}\right)\right)P_{A}\left(t_{A}\right)P_{B}\left(t_{B}\right) & \text{if}\;0\leq \rho_{s}\leq 1\\ \delta \left(S_{A}\left(t_{A}\right)+S_{B}\left(t_{B}\right)-1\right)P_{A}\left(t_{A}\right)P_{B}\left(t_{B}\right) & \text{if}\;-1\leq \rho_{s}\leq 0 \end{array}\right. \end{array}$$

Where δ(x) is the Dirac’s Delta function. Note that, if X denotes either A or B, $S_{X}\left(t_{X}\right)=\int_{t_{X}}^{\infty }P_{X}\left(t\right)dt$ and $D_{X}$ is the cumulative distribution of $P_{X}\left(t\right)$, then $S_{X}\left(t\right)=1-D_{X}\left(t\right)$. It is not difficult to show that the marginal distributions of $P(t_{A},t_{B})$ coincide with $P_{A}\left(t_{A}\right)$ and $P_{B}\left(t_{B}\right)$. We will now use Equation 7, for which we need to integrate Equation 19 in the rectangle $0\leq t_{A}\leq 1$ and $0\leq t_{B}\leq 1$. The term pertaining to the fraction 1−α of independently chosen points $t_{A}$ and $t_{B}$ can be easily integrated to yield

(Equation 20) $$\int_{t_{A}=0}^{t}\int_{t_{B}=0}^{t}P_{A}\left(t_{A}\right)P_{B}\left(t_{B}\right)dt_{A}dt_{B}=\left(1-S_{A}\left(t\right)\right)\left(1-S_{B}\left(t\right)\right)\text{.}$$

To handle the second term we have to consider the positive and negative correlation cases independently.

1) Case $0\leq \rho_{s}\leq 1$

We change variables to $z_{A}=S_{A}\left(t_{A}\right)$ and $z_{B}=S_{B}\left(t_{B}\right)$.

(Equation 21) $$\begin{array}{cc} \int_{t_{A}=0}^{t}\int_{t_{B}=0}^{t}\delta (S_{A}\left(t_{A}\right) & -S_{B}\left(t_{B}\right))\;P_{A}\left(t_{A}\right)P_{B}\left(t_{B}\right)dt_{A}dt_{B}=\\ & =\int_{S_{A}\left(t\right)}^{1}\int_{S_{B}\left(t\right)}^{1}\delta \left(z_{A}-z_{B}\right)dz_{A}dz_{B}=\int_{S_{\max}\left(t\right)}^{1}dz_{\max}=1-S_{\max}\left(t\right)\text{,} \end{array}$$

where we first integrated between the variable $z_{\min}$ such that $S_{\min}\left(t\right)=\min\left(S_{A}\left(t\right),S_{B}\left(t\right)\right)$ to ensure that the first integral yielded 1. Putting together Equation 20 and (Equation 21), (Equation 22) and using Equation 7 we get

(Equation 22) $$\begin{array}{cc} S_{AB}\left(t\right) & =1-\alpha \left(1-S_{\max}\left(t\right)\right)+\left(1-\alpha \right)\left(1-S_{\max}\left(t\right)\right)\left(1-S_{\min}\left(t\right)\right)\\ & =S_{\max}\left(t\right)+S_{\max}\left(t\right)\left(1-S_{\min}\left(t\right)\right)\left(1-\alpha \right)\text{.} \end{array}$$

which coincides with the positive $\rho_{s}$ case of Equation 18.

2) Case $-1\leq \rho_{s}\leq 0$

Starting from the negative $\rho_{s}$ branch of Equation 19 we change variables to $z_{A}=S_{A}\left(t_{A}\right)$ and $z_{B}=1-S_{B}\left(t_{B}\right)$.

(Equation 23) $$\int_{t_{A}=0}^{t}\int_{t_{B}=0}^{t}\delta \left(S_{A}\left(t_{A}\right)+S_{B}\left(t_{B}\right)-1\right)\;P_{A}\left(t_{A}\right)P_{B}\left(t_{B}\right)dt_{A}dt_{B}==\int_{S_{A}\left(t\right)}^{1}\int_{0}^{1-S_{B}\left(t\right)}\delta \left(z_{A}-z_{B}\right)dz_{A}dz_{B}=\int_{S_{A}\left(t\right)}^{1}\int_{0}^{S_{A}\left(t\right)}\delta \left(z_{A}-z_{B}\right)dz_{A}dz_{B}+\int_{S_{A}\left(t\right)}^{1}\int_{S_{A}\left(t\right)}^{1-S_{B}\left(t\right)}\delta \left(z_{A}-z_{B}\right)dz_{A}=\max\left(0,1-S_{A}\left(t\right)-S_{B}\left(t\right)\right)$$

where we put together Equations 20 and 23 and using Equation 7 we get

(Equation 24) $$\begin{array}{cc} S_{AB}\left(t\right) & =1-\alpha \;\max\left(0,1-S_{A}\left(t\right)-S_{B}\left(t\right)\right)+\left(1-\alpha \right)\left(1-S_{A}\left(t\right)-S_{B}\left(t\right)+S_{A}\left(t\right)S_{B}\left(t\right)\right)\\ & =\alpha \;\min\left(1,S_{A}\left(t\right)+S_{B}\left(t\right)\right)+\left(1-\alpha \right)\left(S_{A}\left(t\right)+S_{B}\left(t\right)-S_{A}\left(t\right)S_{B}\left(t\right)\right) \end{array}$$

which coincides with the negative $\rho_{s}$ case of Equation 18.

To see the relation between the Spearman’s correlation $\rho_{s}$ and the parameter α, we consider a cohort of N patients, each one with a pair of survival times $t_{A}$ and $t_{B}$. For each sample we will have the ranking of the survival times which we will call $i_{A}$ and $i_{B}$. Clearly $1\leq i_{A},i_{B}\leq N$.The probability over all possible cohorts of having a patient with a pair of survival times ($t_{A}$, $t_{B}$) will be denoted by $P\left(i_{A},i_{B}\right)$. As before, we will consider a mixture distribution with a fraction 1−α of the patients with random ranks $i_{A}$ and $i_{B}$ (corresponding to a distribution $P_{r}\left(i_{A},i_{B}\right)=\frac{1}{N^{2}}$, and the other fraction of α patients being either perfectly correlated (with distribution $P_{\rho_{s}=1}\left(i_{A},i_{B}\right)=\frac{1}{N}\delta_{i_{A},i_{B}}$) or perfectly anti-correlated (with distribution $P_{\rho_{s}=-1}\left(i_{A},i_{B}\right)=\frac{1}{N}\delta_{N+1-i_{A},i_{B}}$),

(Equation 25) $$P\left(i_{A},i_{B};\rho_{s}\right)=\left(1-\alpha \right)\frac{1}{N^{2}}+\alpha \left\{ \begin{array}{ll} \frac{1}{N}\delta_{i_{A},i_{B}} & \text{if}\;0\leq \rho_{s}\leq 1\\ \frac{1}{N}\delta_{N+1-i_{A},i_{B}} & \text{if}\;-1\leq \rho_{s}\leq 0 \end{array}\right.$$

The Spearman correlation is the rank correlation of the survival times, and as such, it can be computed as

(Equation 26) $$\rho_{s}=\frac{\left\langle i_{A}i_{B}\right\rangle -\left\langle i_{A}\right\rangle \left\langle i_{B}\right\rangle }{\sigma_{i_{A}}\sigma_{i_{B}}}\text{.}$$

where $\delta_{i,j}$ is Kronecker’s delta, $\sigma_{i_{A}}^{2}=\left\langle i_{A}^{2}\right\rangle -{\left\langle i_{A}\right\rangle }^{2}$ and $\sigma_{i_{B}}^{2}=\left\langle i_{B}^{2}\right\rangle -{\left\langle i_{B}\right\rangle }^{2}$. To proceed, we compute $\left\langle i_{A}i_{B}\right\rangle$, $\left\langle i_{A}\right\rangle$ and $\left\langle i_{B}\right\rangle$ using the expression of $P\left(i_{A},i_{B};\rho_{s}\right)$ for positive and negative $\rho_{s}$. Note that the marginal distributions of $i_{A}$ and $i_{B}$ are the same regardless of the sign of $\rho_{s}$, that is $P\left(i_{B}\right)=\frac{1}{N}$, and therefore

(Equation 27) $$\left\langle i\right\rangle :=\left\langle i_{A}\right\rangle =\left\langle i_{B}\right\rangle =\frac{N+1}{2}$$

(Equation 28) $$\left\langle i^{2}\right\rangle :=\left\langle i_{A}^{2}\right\rangle =\left\langle i_{B}^{2}\right\rangle =\frac{\left(N+1\right)\left(2N+1\right)}{6}$$

(Equation 29) $$\sigma_{i}^{2}:=\sigma_{i_{A}}^{2}=\sigma_{i_{B}}^{2}=\frac{\left(N+1\right)\left(N-1\right)}{12}$$

i) Case positive $\rho_{s}$:

In this case

(Equation 30) $$\left\langle i_{A}i_{B}\right\rangle =\left(1-\alpha \right)\frac{1}{N^{2}}\sum_{i_{A}=1}^{N}i_{A}\sum_{i_{B}=1}^{N}i_{B}+\alpha \frac{1}{N}\sum_{i_{A}=1}^{N}i_{A}\sum_{i_{B}=1}^{N}i_{B}\delta_{i_{A},i_{B}}$$

(Equation 31) $$\begin{array}{cc} & =\left(1-\alpha \right){\left(\frac{1}{N}\sum_{i=1}^{N}i\right)}^{2}+\alpha \frac{1}{N}\sum_{i=1}^{N}i^{2} \end{array}$$

(Equation 32) $$\begin{array}{cc} & =\left(1-\alpha \right){\left\langle i\right\rangle }^{2}+\alpha \left\langle i^{2}\right\rangle \end{array}$$

Using Equation 26 we find that

(Equation 33) $$\begin{array}{cc} \rho_{s} & =\frac{\left(1-\alpha \right){\left\langle i\right\rangle }^{2}+\alpha \left\langle i^{2}\right\rangle -{\left\langle i\right\rangle }^{2}}{\sigma_{i}^{2}}=\alpha \frac{\left\langle i^{2}\right\rangle -{\left\langle i\right\rangle }^{2}}{\sigma_{i}^{2}}\\ & =\alpha \text{.} \end{array}$$

ii) Case negative $\rho_{s}$:

In this case

(Equation 34) $$\left\langle i_{A}i_{B}\right\rangle =\left(1-\alpha \right)\frac{1}{N^{2}}\sum_{i_{A}}^{N}i_{A}\sum_{i_{B}=1}^{N}i_{B}+\alpha \frac{1}{N}\sum_{i_{A}=1}^{N}i_{A}\sum_{i_{B}=1}^{N}i_{B}\delta_{N+1-i_{A},i_{B}}$$

(Equation 35) $$\begin{array}{cc} & =\left(1-\alpha \right){\left(\frac{1}{N}\sum_{i=1}^{N}i\right)}^{2}+\alpha \frac{1}{N}\sum_{i=1}^{N}\left(N+1-i\right)i \end{array}$$

(Equation 36) $$\begin{array}{cc} & =\left(1-\alpha \right){\left\langle i\right\rangle }^{2}+\alpha \left(\frac{{\left(N+1\right)}^{2}}{2}-\left\langle i^{2}\right\rangle \right)\\ & =\left(1+\alpha \right){\left\langle i\right\rangle }^{2}-\alpha \left\langle i^{2}\right\rangle \end{array}$$

Using Equation 26 we find that

(Equation 37) $$\begin{array}{cc} \rho_{s} & =\frac{\left(1+\alpha \right){\left\langle i\right\rangle }^{2}-\alpha \left\langle i^{2}\right\rangle -{\left\langle i\right\rangle }^{2}}{\sigma_{i}^{2}}=\alpha \frac{{\left\langle i\right\rangle }^{2}-\left\langle i^{2}\right\rangle }{\sigma_{i}^{2}}\\ & =-\alpha \text{.} \end{array}$$

It follows that for this choice of distribution, the tCDA model is exact and corresponds to $\alpha \left(\rho_{s}\right)=\left|\rho_{s}\right|$.

##### Pairing survival times under two drugs to attain a given Spearman correlation

Given two survival curves corresponding to two monotherapies A and B that are also tested in combination, we would like to have a method to pair samples of survival times in such a way that we have a given Spearman’s correlation. This can be done by drawing N independent samples $t_{A}$ consistent with drug A’s survival curve (note: 1 - survival curve = cumulative distribution). Likewise, we can draw another set of N independent samples $t_{B}$ consistent with drug B’s survival curve and pair these survival times until they have a desired Spearman correlation.

There are many possible ways to reorganize two vectors such that they have a specified correlation while keeping the corresponding marginal distributions. Below we discuss two different ways to accomplish this, but there are many other ways to do it that we have not explored. We believe that each method for randomizing with correlation is equally likely to be the true method by which nature operates, but we are unaware of any way to discern which method may be more likely to be true. Thus, we proceed with the two methods introduced here. One is the numerical implementation of the theoretical joint distribution discussed in the previous section, and as such, it has the advantage that we can use analytical expressions for their implementation. The second method is used as a comparative alternative, to determine the dependence of the results on the specific way of pairing the survival times. We will see that the final conclusions are robust to the specific pairing method used. Both methods start by fixing one vector of times, say $t_{A}$, and shuffle the other vector appropriately until the specified correlation is reached to within a tolerance.

###### The window-swap method

Let us assume that our target correlation $\rho_{s}$ is positive. The method starts with two vectors of times $t_{A,i}$ and $t_{B,i}$ ordered from the minimum values $t_{A,1}$ and $t_{B,1}$, to their maximum values $t_{A,N}$ and $t_{B,N}$, in such a way that their Spearman correlation is 1. To attain our target Spearman correlation we “disorganize” the relative order of the $t_{A}$ and $t_{B}$ vectors in a controlled way. To do that we go through each index i and use a symmetric window of size w around the current index comprising indices ranging from i−w to i+w (if i−w is less than 1, we clamp the minimum at 1; if i+w is larger than N, we clamp the maximum at N). We then choose a random index j in the interval $\left[i-w,i+w\right]$ and swap the values of $t_{B}$ at positions j and i. After visiting all indices we compute the Spearman correlation. If its value is larger than the target one, we restart the process with a larger w (e.g., doubling the previous one, or increasing the previous one to w+1). If the last iteration created a Spearman correlation that is smaller than the target, we go back to the previous iteration and redo the last iteration with a smaller window (e.g. halving the previous one or increasing the previous one to w−1). This process converges when we arrive at a Spearman correlation that differs from the target correlation within a pre-specified tolerance, e.g., 1%. The previous process need only work for positive target correlation values. If our target correlation $\rho_{s}$ is negative, we find the shuffled vector using the absolute value of $\rho_{s}$ and once it converges, we simply flip the shuffled $t_{B}$ vector to get $t_{B}^{\prime }$, the appropriate vector (i.e. reverse order such that $t_{B,i}^{\prime }=t_{B,N+1-i}$).

###### The coin method

The coin method is parametrized by the weight of the coin - the probability α of landing heads. Similar to the window-swap method, this method starts with two vectors of times $t_{A,i}$ and $t_{B,i}$ ordered from the minimum to the maximum values. For each index i in the to-be-shuffled vector, we flip the weighted coin with weight α. If heads, we randomly swap the current value $t_{B,i}$ with the value $t_{B,j}$ at an index j randomly chosen in the interval [1,N] but excluding the current index i. This coin method of simulation is an implementation of the theory presented in the previous section. Therefore the target Spearman correlation, $\rho_{s}$, can directly be used as the weight of the coin according to $\alpha =\left|\rho_{s}\right|$. Just as for the window swap method, we flip the resulted shuffled vector if the desired Spearman’s correlation is negative. Figure S7 exemplifies the results of the previous section in the context of the coin method. Figure S7A shows the results of the coin method to create a joint distribution of survival times for which a target fraction |ρ| of points falls on the orange line (the “ρ=1 line”) where we plot the rank ordered times $t_{A}$ versus the rank ordered $t_{B}$; the remaining 1−|ρ| fraction of points were randomly reordered according to the coin method, but maintaining the marginal distributions of $t_{A}$ and $t_{B}$. For each target ρ, we simulated the joint survival time distribution using the coin method and computed the fraction of points that remained in the “ρ=1 orange line and the Spearman correlation of the simulated data, and plotted them in Figure S7B. As the result of the previous section anticipated, the fraction of points that remained organized in the ρ=1 line is very approximately equal to the measured Spearman correlation. The discrepancies are due to errors due to sample size. These errors would asymptotically decrease as the number of points increase.

###### Survival curves are relatively insensitive to the joint distribution of survival times

Both the coin method and the window-swap method produce joint distributions of survival times that maintain invariant the marginal distributions and have a given Spearman correlation between -1 and 1. These resulting joint distributions, however, could be quite different, especially as the correlation becomes closer to 1 and -1. Figure S8A shows the results of the joint distributions for two simulated monotherapies with Hill-curve shaped marginal distributions and Spearman correlation set to 0.25. We can see that there is a remnant of the sorted times in the coin method, delineating a curve that contains 25% of the points, as we showed above. No such line exists for the window swap method. Outside of this line, the remaining points in the coin-method-based joint distribution are independently distributed. In the window-swap-based joint distribution (orange points) all subsets of points are dependent with a Spearman correlation of 0.25. Figure S8B shows the resulting survival curve corresponing to patients treated with the combination by applying taking the maximum of the survival times under each monotherapy for each simulated patient using the coin simulation (black line) and the window-swap method (orange line). It can be clearly seen that the survival distribution in response to the combination is insensitive to the background joint distribution of survival times.

Figures S8C and S8D show similar results as Figures S8A and S8B but using the actual monotherapy survival distributions observed in a clinical trial with Erlotinib and Bevacizumab. Figure S8D shows subtle differences between the coin and window-swap methods, especially for longer survival times. Despite these differences, the two curves are quantitatively very similar, showing that the joint distribution of survival times has at worst a mild effect in the survival distribution in response to the combination treatment resulting from the CDA approach.

##### Fitting the tCDA model to the data

Given the survival probabilities of the monotherapies and their combination, we need to determine how well the tCDA model fits the combination data. For that, we need to find the value of $\rho_{s}$ in the interval [−1,1] that results in the best tCDA approximation to the actual combination survival curve. To do so, we can use one of the tCDA implementations (analytical model, coin-based simulation, window swap simulation).

In this work, we deal exclusively with progression-free survival (PFS) curves. But, our methods and approach can be used for any sort of survival metric such as Overall Survival (OS) too.

We estimate the PFS in regular time intervals (we choose 0.05 month intervals) using the scraped raw data via linear interpolation. Next, we find the maximum time point for each trial that we have data for. Then, we compute the minimum of these times across all trials in the combination. We filter all (time, PFS) points with time coordinate values greater than this minimum to ensure that missing values do not interfere with the fitting process.

Now, let us describe fitting using the tCDA analytical model. First, for a given combination, we must assemble progression-free survival tables formatted according to the preceding paragraph. We use Equation 18, and make the simplifying assumption that $\alpha \left(\rho_{s}\right)=|\rho_{s}|$. We nominate 200 equally spaced candidate values of $\rho_{s}$ on the interval [−1,1] and for each candidate $\rho_{s}$ we compute its predicted combination survival curve, choosing the $\rho_{s}$ that minimizes the root mean square error (RMSE) between the observed combination and predicted combination.

As a test that the our fitting procedure captures the right parameter when it should, we simulate survival times for each patient treated with a drug combination using two monotherapies whose survival curves are assumed to follow Hill curves. We then use the coin model to set a correlation between the survival times and simulate the survival in response to the combination as the maximum of the survivals of the monotherapies for each patient. The resulting survival curves for 4 values of correlations are shown as solid blue curves in Figure S9A. We then fit the best parameter α for the tCDA model from the monotherapies for each correlation shown in the subfigures, and find that the tCDA model fits the simulated survival under the combination treatment extremely well (dashed line in the figure). Furthermore, the fitted values of α resulting from fitting the tCDA model match extremely well the target correlation used in the coin method in the whole range of correlations between -1 and 1 ($R^{2}$ =0.9998) as shown in Figure S9B. This shouldn’t surprise us, as in the previous section we proved that the coin method results in a joint distribution of survival times for which the tCDA model is the exact analytical solution. To show the robustness of the tCDA model to capture the underlying correlation, we followed the same procedure used in Figure S9 but instead of using the coin method for correlating the joint distribution we used the window swap method. Figure S10 shows the results. Again the survival distribution for the different imposed correlations (blue solid lines in Figure S10A is captured very well by the tCDA model with fitted parameter α. Figure S10B shows that the value of α tracks remarkably well the target correlation of the simulations, with an $R^{2}$ =0.991. The results shown in this Figure are remarkable as they show that the analytical tCDA model very closely follow the correlation of the joint distribution even when the joint distribution is not the one underlying the analytical model. The conclusion is that the tCDA model is relatively insensitive to the details of the joint distribution of survival times.

We will now describe fitting using the tCDA simulation methods (either the coin or window swap method). For a given combination, there are two individual monotherapies for which we collect data on. For each of these trials, we have the PFS curve and, therefore, the cumulative distribution (1 - PFS curve is the cumulative distribution). We now sample survival times for hypothetical patients from the cumulative distribution via an inverse transform. The number of hypothetical patients should be the same for each trial in the combination and large (we choose it to be 4 times the number of time points for which we have data for). In practice, it can be set rather arbitrarily. Next, we sort the sampled hypothetical patient PFS times for either monotherapy in decreasing order. Concurrently, we also format the data according to the instructions detailed two paragraphs above. We nominate 200 equally spaced candidate values of $\rho_{s}$ on the interval [−1,1]. For each candidate $\rho_{s}$, we shuffle the paired survival times according to the desired simulation method (i.e., window swap or coin) such that the Spearman’s correlation between the two vectors of monotherapy PFS times are $\rho_{s}$. We now have a matrix where each row corresponds to a hypothetical patient and the two columns corresponds to the patients’ PFS time under either monotherapy. For each patient, we take the maximum survival time across monotherapies and refer to it as the patients’ combination PFS time under the tCDA simulation method. Finally, we can use this distribution of tCDA combination survival times to compute the tCDA estimated PFS survival curve for the candidate $\rho_{s}$. To compare this estimate to the observed combination PFS curve, we linearly interpolate the tCDA combination estimate on the same time intervals as done for the observed combination. We then compute the RMSE between the tCDA and observed combination and choose the $\rho_{s}$ that minimizes this error.

The confidence interval for $\rho_{s}$ in the tCDA model (analytical and simulation models) is computed via a bootstrap approach. We use the empirical inverse transform method to generate new survival times that are distributed according to the original survival curve. The cumulative distribution of the original survival curve is simply 1 minus the original survival curve. We generate resampled data for both of the monotherapies and the combination. To ensure the bootstrapped trial’s variability is kept consistent with the original trial, the number of samples taken from the original survival curve is set to be the number of patients in the corresponding clinical trial from where the data was obtained. Then an optimal ρ is computed with the resampled data according to the fitting process of the desired model in a process that is repeated 5000 times to construct the appropriate 95% confidence interval. Specifically, we took the 2.5% and 97.5% quantiles of the empirical distribution for ρ’s to be the extremes of the 95% confidence interval.

##### Detailed examination of the tCDA model with Herceptin and chemotherapy combination trial data

We next examine the combination of chemotherapy and Trastuzumab (Herceptin). Chemotherapy consisted of anthracycline plus cyclophosphamide for patients who had never received anthracycline before or paclitaxel for patients who had received adjuvant anthracycline.^24^ This therapy is a current treatment for HER-2 overexpressing breast cancers. The Tratuzumab and chemotherapy combination shows a clinical benefit over either individual monotherapy with respect to progression-free survival (PFS) time (Figure S3A). At around 12 months, the benefit of the combination reduces to that of the Trastuzumab treated group. As shown earlier, the tCDA model predicts the PFS curve $S_{AB}\left(t\right)$ of the combination of the two drugs A and B (in this case chemotherapy and Trastuzumab) based on the PFS curves of each of the monotherapies $S_{A}\left(t\right)$ and $S_{B}\left(t\right)$ in the form prescribed by the mathematical expression

(Equation 38) $$\begin{array}{cc} S_{AB}\left(t,\rho \right)= & \\ & =\left\{ \begin{array}{ll} S_{\max}\left(t\right)+S_{\min}\left(t\right)\left(1-S_{\max}\left(t\right)\right)\left(1-\rho \right) & \text{if}\;0\leq \rho \leq 1\\ \left(S_{A}\left(t\right)+S_{B}\left(t\right)-S_{A}\left(t\right)S_{B}\left(t\right)\right)\left(1-\left|\rho \right|\right)+\min\left(1,S_{A}\left(t\right)+S_{B}\left(t\right)\right)\left|\rho \right| & \text{if}\;-1\leq \rho \leq 0 \end{array}\right. \end{array}$$

where $S_{\max}\left(t\right)=\max\left(S_{A}\left(t\right),S_{B}\left(t\right)\right)$, $S_{\min}\left(t\right)=\min\left(S_{A}\left(t\right),S_{B}\left(t\right)\right)$, and ρ is the correlation over all patients of the times that each patient would have survived if treated with drug A and B independently. As we vary the parameter ρ in Equation 38 using as $S_{A}\left(t\right)$ and $S_{B}\left(t\right)$ the observed PFS curves of the chemotherapy and Trastuzumab respectively, we span the range of the tCDA predictions forming the cone of possibilities shown in Figure S3B, where each color corresponds to one value of correlation.

Figure S3C shows different simulations (using what we called the ”Coin method” described earlier in the Supplement) of survival times for different overall correlations in the patient population. Each point in each subpanel of Figure S3C represents a simulated patient, and the y-axis and x-axis values are the survival times that a given patient would have survived if treated only with Trastuzumab or chemotherapy respectively. The marginal distribution of x-axis and y-axis values in all subfigures follow $S_{A}\left(t\right)$ and $S_{B}\left(t\right)$ curves respectively, and the correlations alluded to in the figure are Spearman’s rank correlations. A value of Spearman’s correlation equal to 1 traces the “Time Equivalence Curve” between Trastuzumab and chemotherapy while a value of -1 associates the survival times of patients to drugs Trastuzumab and chemotherapy in reverse order. A Spearman’s correlation of zero assigns survival times to Trastuzumab and chemotherapy independently of one another. From this joint distribution of patient survival times and by taking the maximum value at each ordered pair, we can construct the empirical estimate of the PFS curve under tCDA model at a given Spearman’s correlation. This simulation process is mathematically equivalent to the closed-form expression given in Equation 38 which we use throughout the paper. We will estimate the tCDA predicted PFS curve of the combination by varying the parameter ρ between [−1,1] in the analytical model in Equation 38 and choosing the value of ρ that minimizes the root mean square error (RMSE) between the estimated and observed combination PFS curves.

Returning to the Trastuzumab and chemotherapy trial data, the optimal estimate for the Spearman’s correlation is 0.03 and its corresponding 95% confidence interval is [−0.07,0.13]. The confidence intervals are computed via a bootstrap approach where the data is resampled many times to generate an empirical distribution of correlation estimates as described earlier. We can observe that the resulting estimate for the combination PFS curve under tCDA follows the true combination rather well (Figure S3D). Empirically, using a Kolmogorov-Smirnov test we fail to reject the null hypothesis that the tCDA model sufficiently describes the observed combination (p-value of 0.46). In this sense, it can be hypothesized that this combination is not inconsistent with the tCDA independent action assumption.

Interestingly, a ρ=0, as we approximately have in this case, reduces Equation 38 into a mathematical form analogous to that of Bliss independence used in the dose domain, namely $S_{AB}\left(t,\rho =0\right)=S_{\max}\left(t\right)+S_{\min}\left(t\right)\left(1-S\;\max\left(t\right)\right)$. A zero Spearman’s correlation indicates that the survival times that can be attributed to each of the treatments are independent of each other in the population. This suggests that in the absence of a stratification strategy to separate metastatic HER-2 overexpressing breast cancer patients into those that respond better to Trastuzumab and those that respond better to chemotherapy, it is better to administer the combination to all such patients rather than trying to assign each patient to the monotherapy that will work best for them. In this scenario, when we are fundamentally unable to decide for a given patient whether Trastuzumab or chemotherapy is better for them and in order to reduce the guesswork in the process, the best strategy is to give the combination for each patient.

##### Additional examples of tCDA model applied to combination trial data

Figures S4–S6 show several examples of combinations for which the tCDA model yield different Spearman’s correlations include 5-FU and Oxaliplatin in advanced pancreatic cancer, Interferon Alfa and Temsirolimus in advanced renal cell carcinoma, Irinotecan Bevacizumab and Panitunumab in advanced colorectal cancer, and other combination chemotherapy regimens. Of course, we must be sufficiently certain that the tCDA model describes well the effect of the combination, and even in that case we must be careful to not over-interpret the meaning of the Spearman’s correlation resulting from the tCDA model fit.

#### dCDA model

##### Mathematical details

Independent drug action has been mostly explored in the in the context of temporal responses such PFS or OS in clinical trials or pre-clinical research.^7^^,^^25^ In this section we investigate the principle of independent drug action at the level of cell cultures and in dose space. To do so we apply the same principles that were applied in previous sections but rather than asking for the survival time of a patient under each of two drugs, we will ask for the survival of a cell to each of two drugs at their respective concentrations and after a fixed time has elapsed since treatment. The assumption in what follows is that each cell will die because of the effect of the most efficacious drug for that cell, and like we did before with the survival time for each patient, we will consider that the doses at which a cell dies with one or another drug are correlated. This correlation could be due to confounding variables. In cell cultures we can think that specific characteristics of a cell such its number of mitochondria,^20^ its size, its protein content, etc, could be a confounder. We will call this application of correlated response of cells in dose space as Dose Correlated Drug Action, or dCDA.

Like tCDA, dCDA is an independent drug action model for the action of drug combination on cells cultures when studying their dose response curves. There is a body of literature that addresses the problem of predicting the dose-response of a drug combination given the dose responses of the constituent monotherapies under the baseline assumptions of independence or additivity.^26^ The most used baseline models are Bliss’ independent joint action model^8^ and Loewe’s dose additivity principle.^27^ The latter can be transformed into a quantitative measure called the Combination Index,^28^ and was the basis of the more general principle of dose equivalence.^29^ There are other methods developed for drug combination models such as the Highest Single Agent,^30^ and some frameworks for their integration into a unified framework.^31^^,^^32^ The dCDA starting point is the quantification of killing effect of a therapy on a cancer cell culture using the dose response curve that estimates the survival of a cell culture with respect to a control population. The dCDA model postulates that the effect of two therapies in combination is the same as the effect of the most effective of the two therapies at the single cell level. If we knew *a priori* which of two therapies yields better results for a given cell in a culture, dCDA postulates that the effect of administering the best monotherapy for that cell would be the same as the effect of administering the combination.

The doses at which cells die vary from cell to cell due to different states in which cells could be, such as abundance of key proteins, number of mitochondria, its size, etc. dCDA assumes that the lethal doses on the same cell in response to each of two drugs could be correlated.

The dCDA framework provides a baseline hypothesis for independent drug action that can be used as a null hypothesis in a statistical test to classify combinations as consistent with dCDA or not consistent with dCDA. Non-dCDA combinations that are of particular interest are antagonistic or synergistic combinations.

##### Notation

For each cell in a cell culture, we will denote by $\delta_{A}$ and $\delta_{B}$ the doses at which the cell would be dead at a given time after treatment (typical assays measure viability at a time between 24h to 72h). If treated with drug X (X=A or B) at dose $D_{X}$, a cell with a lethal dose of $\delta_{X}$ will be found dead at time T if the effective dose of drug X was larger than $\delta_{X}$. Under the dCDA model, if a cell with lethal doses $\left(\delta_{A},\delta_{B}\right)$ is treated with both drugs at doses $\left(D_{A},D_{B}\right)$ then the cell will die if either $\delta_{A}<D_{A}^{\prime }$ or $\delta_{B}<D_{B}^{\prime }$. Here $D_{A}^{\prime }$ is the effective dose of A, enhanced by the fact that the culture is also treated with drug B. Similarly, $D_{B}^{\prime }$ is the effective dose of B, enhanced by the fact that the culture is also treated with drug B. We will discuss in a subsequent section how to calculate $D_{A}^{\prime }$ and $D_{B}^{\prime }$. For a cell to survive, both lethal doses have to be larger than the equivalent treatment doses.

The dose response curve that a generic cell in a cell culture survives at dose $D_{A}$, $D_{B}$ under treatments A, B and combination AB will be denoted by $V_{A}\left(D_{A}\right)$, $V_{B}\left(D_{B}\right)$ and $V_{AB}\left(D_{A},D_{B}\right)$, and can be formally expressed as

(Equation 39) $$V_{A}\left(D_{A}\right)=\text{Prob}\left(\delta_{A}>D_{A}\right)$$

(Equation 40) $$V_{B}\left(D_{B}\right)=\text{Prob}\left(\delta_{B}>D_{B}\right)$$

(Equation 41) $$V_{AB}\left(D_{A},D_{B}\right)=\text{Prob}\left(\delta_{A}>D_{A}^{\prime },\delta_{B}>D_{B}^{\prime }\right)$$

In practice the probabilities are computed as fractions in a cell culture. For example, the $V_{A}\left(D_{A}\right)$ is the fraction of cells in culture that are still alive at dose $D_{A}$ and therefore is the fraction of cells whose lethal dose $\delta_{A}$ is larger than $D_{A}$.

The fraction of cells that die is call the inhibited fraction, and is equal to 1 minus the viability, that is:

(Equation 42) $$I_{A}\left(D_{A}\right)=1-V_{A}\left(D_{A}\right)=\text{Prob}\left(\delta_{A}<D_{A}\right)$$

(Equation 43) $$I_{B}\left(D_{B}\right)=1-V_{B}\left(D_{B}\right)=\text{Prob}\left(\delta_{B}<D_{B}\right)$$

(Equation 44) $$I_{AB}\left(D_{A},D_{B}\right)=1-V_{AB}\left(D_{A},D_{B}\right)=\text{Prob}\left(\delta_{A}<D_{A}^{\prime }\;\text{OR}\;\delta_{B}<D_{B}^{\prime }\right)$$

(Equation 45) $$\text{Prob}\left(\delta_{A}<D_{A}^{'}\;\text{OR}\;\delta_{B}<D_{B}^{'}\right)\text{ = 1-Prob}\left(\delta_{A}\geq D_{A}^{'}\;\text{AND}\;\delta_{B}\geq D_{B}^{'}\right)$$

The inhibited fractions under drug A or B is equal to the cumulative distribution of the lethal times under those drugs in a cell culture. Therefore, the inhibited fraction or the viability of a cell culture under the effect of drug X can be written in terms of the probability density $P\left(\delta_{X}\right)$ that a cell has lethal dose $\delta_{X}$ under treatment X as

(Equation 46) $$I_{X}\left(D_{X}\right)=\int_{0}^{D_{X}}P_{X}\left(\delta \right)d\delta$$

To measure the correlation between lethal doses $\delta_{A}$ and $\delta_{B}$ we will use the Spearman’s rank correlation. This is computed by ranking the $\delta_{A}$’s and $\delta_{B}$’s in a cell culture and computing the correlation between the rankings of $\delta_{A}$ and $\delta_{B}$ for the same cell in the cell culture.

##### Formulation of the Dose Correlated Drug Action model

In this section, we want to derive expressions for the viability of a cell culture under treatment with both drugs A and B at doses $D_{A}$ and $D_{B}$ in terms of the viabilities of the monotherapies. To proceed we consider separately the correlated and the uncorrelated cases.

##### Uncorrelated case

We will consider that the two lethal doses $\delta_{A}$ and $\delta_{B}$ for treatments with drugs A and B are uncorrelated because they are statistically independent. (In the general case, the drugs could be uncorrelated but statistically dependent). In this case, the equivalent doses $D_{A}^{\prime }$ and $D_{B}^{\prime }$ for A and B are equal to the given doses $D_{A}$ and $D_{B}$ because the presence of A (resp. B) does not affect A (resp. B) in any way, and $\text{Prob}\left(\delta_{A}\geq D_{A}\;\text{AND}\;\delta_{B}\geq D_{B}\right)=\text{Prob}\left(\delta_{A}\geq D_{A}\right)\text{Prob}\left(\delta_{B}\geq D_{B}\right)$, which leads to

(Equation 47) $$V_{AB}\left(D_{A},D_{B},\rho_{s}=0\right)=V_{A}\left(D_{A}\right)V_{B}\left(D_{B}\right)\text{,}$$

Note that in this case, the viability under the two drugs is smaller than the viability under either of the single therapies. If we express the Equation 47 in terms of the Inhibited fraction, we will find the relation $I_{AB}\left(D_{A},D_{B},\rho_{s}=0\right)=I_{A}\left(D_{A}\right)+I_{B}\left(D_{B}\right)-I_{A}\left(D_{A}\right)I_{B}\left(D_{B}\right)$ which has the same expression as Equation 8. The reason why the equation for the survival probability in tCDA is formally the same as the inhibition fraction in dCDA is because the former results from a logical OR condition (patients are alive if $t_{A}>t$ OR $t_{B}>t$) whereas in the latter it is the probability of inhibition what follows from an OR condition (cells die if $\delta_{A}<D_{A}$ OR $\delta_{B}<D_{B}$).

##### Positively correlated case

Let us consider the extreme case in which for any given cell, the lethal dose $\delta_{A}$ completely determines the lethal dose $\delta_{B}$, in such a way that the Spearman correlation between the lethal doses under A and B treatments of all cells in a culture is 1. That means that the order of lethal doses of cells under treatment A is the same as the order under treatment B. Take now a cell with lethal doses $\delta_{A}$ and $\delta_{B}$. The fraction $V_{A}\left(\delta_{A}\right)$ of cells that died at dose $D_{A}=\delta_{A}$ should be the same as the fraction $V_{B}\left(\delta_{B}\right)$ of cells that died at dose $\delta_{B}$ under treatment B. Therefore when the Spearman correlation between lethal doses is $\rho_{s}=1$, we have that $V_{A}\left(\delta_{A}\right)=V_{B}\left(\delta_{B}\right)$, from where we have the relationship

(Equation 48) $$\delta_{B}=V_{B}^{-1}\left(V_{A}\left(\delta_{A}\right)\right):=f\left(\delta_{A}\right)\text{,}$$

(Equation 49) $$\delta_{A}=V_{A}^{-1}\left(V_{B}\left(\delta_{B}\right)\right):=g\left(\delta_{B}\right)=f^{-1}\left(\delta_{B}\right)\text{.}$$

Note that the functions $f\left(\delta_{A}\right)$ and $g\left(\delta_{B}\right)$ are increasing functions of $\delta_{A}$ and $\delta_{B}$ respectively, because the viabilities $V_{A}$ and $V_{B}$ and their inverses $V_{B}^{-1}$ and $V_{A}^{-1}$ are decreasing functions of their arguments (the number of surviving cells decreases when we augment the dose of a treatment). Relations (Equation 48), (Equation 49) express what is known as the dose equivalent model, and was developed^29^ in part to account for what is called as sham combination principle^33^ which postulates that a combination of a drug with itself should be additive. In our case, the dose equivalence principle appears very naturally in the limiting case of perfectly correlated lethal doses.

It was mentioned earlier that the presence of drug B at dose $D_{B}$ in the presence of treatment A may enhance the dose of A, $D_{A}$ into and effective dose $D_{A}^{'}$. Symmetrically, the effective dose of B will be enhanced by A from $D_{B}$ to $D_{B}^{\prime }$. We will assume that this enhancement is a function of the correlation between doses, in such way that

(Equation 50) $$D_{A}^{\prime }=D_{A}+\beta \left(\rho_{s}\right)g\left(D_{B}\right)\text{,}$$

(Equation 51) $$D_{B}^{\prime }=D_{B}+\gamma \left(\rho_{s}\right)f\left(D_{A}\right)\text{,}$$

where $\beta \left(\rho_{s}\right)$ and $\gamma \left(\rho_{s}\right)$ control the enhancement of dose of one drug due to the other drug. These functions are unknown *a priori* but we only need to know them in the extreme cases of $\rho_{s}$ = 1, 0 and -1. We can find a criterion to find the values of these functions at $\rho_{s}$ =1, by requiring that dCDA be compatible with sham combinations, that is combination of a drug with itself. In fact when we “combine” a drug with itself, drug B *is* drug A, and therefore for any given cell $\delta_{B}=\delta_{A}$ and the correlation is 1. As in this case the addition of more of the same drug should result in adding the doses, and both $f\left(D_{A}\right)=D_{A}$ and $g\left(D_{B}\right)=D_{B}$, it follows that sham combination compliance requires that β(1)=1 and γ(1)=1. The case $\rho_{s}=0$ is the other extreme in which there is no effect whatsoever direct or indirect of drug A on drug B, and therefore, the presence of one does not change the effective dose of the other, which suggests that β(0)=0 and γ(0)=0. The corner case of $\rho_{s}=-1$ is unusual and it is unclear what the values of these functions should be. In the absence of a clear criterion we will assume β(−1)=0 and γ(−1)=0.

Coming back to $\rho_{s}=1$ we can compute the viability under the combination AB. First we note that we can express $\text{Prob}\left(\delta_{A}\leq D_{A}^{\prime }\;\text{AND}\;\delta_{B}\leq D^{\prime }B\right)$ in terms of conditional probabilities as

(Equation 52) $$\text{Prob}\left(\delta_{A}\geq D_{A}^{\prime }\;\text{AND}\;\delta_{B}\geq D_{B}^{\prime }\right)=\text{Prob}\left(\delta_{A}\geq D_{A}^{\prime }|\delta_{B}\geq D_{B}^{\prime }\right)\text{Prob}\left(\delta_{B}\geq D_{B}^{\prime }\right)$$

(Equation 53) $$\begin{array}{cc} & =\text{Prob}\left(\delta_{B}\geq D_{B}^{\prime }|\delta_{A}\geq D_{A}^{\prime }\right)\text{Prob}\left(\delta_{A}\geq D_{A}^{\prime }\right)\text{.} \end{array}$$

Suppose now that $V_{B}\left(D_{B}^{\prime }\right)<V_{A}\left(D_{A}^{\prime }\right)$. This means that at these doses, there are more cancer cells in culture that died with the equivalent dose of drug B than with the equivalent dose of drug A. It also means that the equivalent dose $D_{A}^{\prime }$ is smaller than the equivalent dose of $g\left(D_{B}^{\prime }\right)$, that is $D_{A}^{\prime }<g\left(D_{B}^{\prime }\right)$. Because the order of lethal doses of the cells in culture is the same under both drugs, then all the cells that survived under the effective dose of B, have lethal doses $\delta_{A}$ larger than $g\left(D_{B}^{\prime }\right)$. Therefore, $D_{A}^{\prime }<g\left(D_{B}^{\prime }\right)<\delta_{B}$. It follows that for $V_{B}\left(D_{B}^{\prime }\right)<V_{A}\left(D_{A}^{\prime }\right)$, $\text{Prob}\left(\delta_{A}\geq D_{A}^{\prime }|\delta_{B}\geq D_{B}^{\prime }\right)=1$. For $V_{A}\left(D_{A}^{\prime }\right)<V_{B}\left(D_{B}^{\prime }\right)$ a symmetric argument leads to $\text{Prob}\left(\delta_{B}\geq D_{B}^{\prime }|\delta_{A}\geq D_{A}^{\prime }\right)=1$. Putting these results together, we have that when there is perfect and positive Spearman correlation, the viability of the combination can be written as

(Equation 54) $$V_{AB}\left(D_{A},D_{B},\rho_{s}=1\right)=\min\left(V_{A}\left(D_{A}^{\prime }\right),V_{B}\left(D_{B}^{\prime }\right)\right)\text{.}$$

For an alternative derivation of this result, we can start from the joint probability distribution

$$P\left(\delta_{A},\delta_{B}\right)=\delta \left(V_{A}\left(\delta_{A}\right)-V_{B}\left(\delta_{B}\right)\right)P_{A}\left(\delta_{A}\right)P_{B}\left(\delta_{B}\right)\text{,}$$

and compute $V_{AB}$ as

(Equation 55) $$V_{AB}\left(D_{A}^{\prime },D_{B}^{\prime }\right)=\int_{D_{A}^{\prime }}^{\infty }\int_{D_{B}^{\prime }}^{\infty }P\left(\delta_{A},\delta_{B}\right)d\delta_{A}d\delta_{B}$$

(Equation 56) $$\begin{array}{cc} & =\int_{V_{A}\left(D_{A}^{\prime }\right)}^{1}\int_{V_{A}\left(D_{B}^{\prime }\right)}^{1}\delta \left(z_{A}-z_{B}\right)dz_{A}dz_{B} \end{array}$$

which leads to Equation 54. Equation 54 is known in the literature as the Higher Single Agent model,^30^ which here results naturally from the fully correlated case of dCDA. However here the arguments of the dose response curves $V_{A}$ and $V_{B}$ are the effective doses of the drugs.

When $\delta_{A}$ and $\delta_{B}$ are uncorrelated and independent, we have that $\text{Prob}\left(\delta_{A}\geq D_{A}^{\prime }|\delta_{B}\geq D_{B}^{\prime }\right)=\text{Prob}\left(\delta_{A}\geq D_{A}\right)$ and symmetrically, $\text{Prob}\left(\delta_{B}\geq D_{B}^{\prime }|\delta_{A}\geq D_{A}^{\prime }\right)=\text{Prob}\left(\delta_{B}\geq D_{B}\right)$, where we used that for $\rho_{s}=0,\;D_{A}^{'}=D_{A}$ and $D_{B}^{\prime }=D_{B}$. In the intermediate case in which we have some correlation $\rho_{s}$, we will interpolate the conditional probabilities in such a way that the completely correlated and uncorrelated case case match the extreme cases as follows

(Equation 57) $$\text{Prob}\left(\delta_{A}\geq D_{A}^{\prime }|\delta_{B}\geq D_{B}^{\prime }\right)=\alpha \left(\rho_{s}\right)+\left(1-\alpha \left(\rho_{s}\right)\right)\text{Prob}\left(\delta_{A}\geq D_{A}^{\prime }\right)\;\text{if}\;V_{B}\left(D_{B}^{\prime }\right)<V_{A}\left(D_{A}^{\prime }\right)$$

(Equation 58) $$\text{Prob}\left(\delta_{B}\geq D_{B}^{\prime }|\delta_{A}\geq D_{A}^{\prime }\right)=\alpha \left(\rho_{s}\right)+\left(1-\alpha \left(\rho_{s}\right)\right)\text{Prob}\left(\delta_{B}\geq D_{B}^{\prime }\right)\;\text{if}\;V_{B}\left(D_{B}^{\prime }\right)\geq V_{A}\left(D_{A}^{\prime }\right)$$

where $\alpha \left(\rho_{s}=0\right)=0$, $\alpha \left(\rho_{s}=1\right)=1$ and the specific functional form in the range $0\leq \rho_{s}\leq 1$ will depend on the joint distribution of lethal doses.

Replacing these results in Equation 52 and Equation 53 and using Equation 41 we find that the viability under the combination AB in the case of $0\leq \rho_{s}\leq 1$ for the dCDA model is

(Equation 59) $$\begin{array}{cc} V_{AB}\left(D_{A},D_{B},0\leq \rho_{s}\leq 1\right) & =V_{A}\left(D_{A}\right)V_{B}\left(D_{B}\right)\left(1-\alpha \left(\rho_{s}\right)\right)\\ & +\left\{ \begin{array}{cc} V_{A}\left(D_{A}+g\left(D_{B}\right)\right)\alpha \left(\rho_{s}\right) & \text{if}\;V_{A}\left(D_{A}+g\left(D_{B}\right)\right)<V_{B}\left(D_{B}+f\left(D_{A}\right)\right)\\ V_{B}\left(D_{B}+f\left(D_{A}\right)\right)\alpha \left(\rho_{s}\right) & \text{if}\;V_{A}\left(D_{A}+g\left(D_{B}\right)\right)\geq V_{B}\left(D_{B}+f\left(D_{A}\right)\right). \end{array}\right. \end{array}$$

##### Negatively correlated case

Let us consider the other extreme case in which for any given cell the lethal dose $\delta_{A}$ completely determines the lethal dose $\delta_{B}$, in such a way that the Spearman correlation between lethal doses under A and B treatments of all cells is -1. That means that the order of lethal doses of cells under treatment A is opposite to the order under treatment B. Take now a cell with lethal doses $\delta_{A}$ and $\delta_{B}$. The fraction $V_{A}\left(\delta_{A}\right)$ of cells that have higher lethal doses than $\delta_{A}$, should be the same as the fraction $1-V_{B}\left(\delta_{B}\right)$ of cells that had smaller lethal doses than $\delta_{B}$. Therefore when the Spearman correlation between lethal doses is $\rho_{s}=-1$, we have that (see Figure )

(Equation 60) $$\delta_{B}=V_{B}^{-1}\left(1-V_{A}\left(\delta_{A}\right)\right):=h\left(\delta_{A}\right)\text{.}$$

In this extreme case we can compute the survival time under the combination AB. Recall that we assumed that in this case $D_{A}^{\prime }=D_{A}$ and $D_{B}^{\prime }=D_{B}$. First we note that there for every dose $D_{A}$ of drug A, there is a dose $h\left(D_{A}\right)$ of drug B such that none the cells that have lethal doses larger than $D_{A}$ (and therefore survived drug A), have lethal doses higher than $h\left(D_{A}\right)$ (and therefore survived B). Therefore for $V_{AB}\left(D_{A},h\left(D_{A}\right)\right)=0$ Figure S2 shows that for $D_{B}<h\left(D_{A}\right)$, the fraction of cells that survived doses $D_{A}$ and $D_{B}$ under A or B is equal to $V_{A}\left(D_{A}\right)+V_{B}\left(D_{B}\right)-1$. Figure S2 also shows that for $D_{B}>h\left(D_{A}\right)$, no cell can survive both $D_{A}$ and $D_{B}$. Therefore for $\rho_{s}=-1$, we have that

(Equation 61) $$V_{AB}\left(D_{A},D_{B},\rho_{s}=-1\right)=\left\{ \begin{array}{ll} 0 & \text{if}\;V_{A}\left(D_{A}\right)+V_{B}\left(D_{B}\right)<1\\ V_{A}\left(D_{A}\right)+V_{B}\left(D_{B}\right)-1 & \text{if}\;V_{A}\left(D_{A}\right)+V_{B}\left(D_{B}\right)\geq 1 \end{array}\right.$$

(Equation 62) $$\begin{array}{cc} & =\max\left(0,V_{A}\left(D_{A}\right)+V_{B}\left(D_{B}\right)-1\right) \end{array}\text{.}$$

In the range of negative correlations between -1 and 0, we can interpolate between the cases of $\rho_{s}=0$ and $\rho_{s}=-1$, which yields

(Equation 63) $$\begin{array}{cc} V_{AB}\left(D_{A},D_{B},-1\leq \rho_{s}\leq 0\right) & =V_{A}\left(D_{A}\right)V_{B}\left(D_{B}\right)\left(1-\alpha \left(\rho_{s}\right)\right)\\ & +\alpha \left(\rho_{s}\right)\max\left(0,V_{A}\left(D_{A}\right)+V_{B}\left(D_{B}\right)-1\right) \end{array}$$

where $\alpha \left(\rho_{s}=0\right)=0$, $\alpha \left(\rho_{s}=-1\right)=1$, and the specific dependence of $\alpha \left(\rho_{s}\right)$ on $\rho_{s}$ depends on the joint distributions of lethal doses $\delta_{A}$ and $\delta_{B}$.

##### Integrated expression for the viability of cells under the dCDA model

The formulas of the previous subsection can be integrated in one simple integrated formula. The expression for the viability under the dCDA model can be written as:

(Equation 64) $$\begin{array}{cc} V_{AB}\left(D_{A},D_{B},\rho_{s}\right) & =\left(1-\alpha \left(\rho_{s}\right)\right)V_{A}\left(D_{A}\right)V_{B}\left(D_{B}\right)\\ & +\alpha \left(\rho_{s}\right)\left\{ \begin{array}{ll} \min(V_{A}\left(D_{A}+g\left(D_{B}\right),V_{B}\left(D_{B}+f\left(D_{A}\right)\right)\right) & \text{if}\;0\leq \rho_{s}\leq 1\\ \max\left(0,V_{A}\left(D_{A}\right)+V_{B}\left(D_{B}\right)-1\right) & \text{if}\;-1\leq \rho_{s}\leq 0 \end{array}\right. \end{array}$$

The model is not complete until we specify the function $\alpha \left(\rho_{s}\right)$, which is unknown except in the border cases $\rho_{s}=$-1, 0, 1 for which $\alpha \left(\rho_{s}\right)$ = is 1, 0, 1. As an approximation, we can assume that $\alpha \left(\rho_{s}\right)=\left|\rho_{s}\right|$. In the next section we will show that this is indeed the correct function for a particular joint distribution for $\delta_{A}$ and $\delta_{B}$.

##### There is a joint distribution of lethal doses for which $\alpha \left(\rho_{s}\right)=\left|\rho_{s}\right|$

There are many possible joint distributions of $\delta_{A}$ and $\delta_{B}$ that yield the same Spearman correlation, and for each such distribution there may be a different function $\alpha \left(\rho_{s}\right)$. Therefore from the perspective of defining uniquely the dCDA model, it is necessary to specify the joint distribution of lethal doses. In this section we will choose such a distribution and show that for it, $\alpha \left(\rho_{s}\right)=\left|\rho_{s}\right|$, which we will call the dCDA joint lethal doses probability density, or dCDA distribution for short.

This distribution can be thought of as a mixture distribution where a fraction 0≤α≤1 of ($\delta_{A}$, $\delta_{B}$) pairs are drawn from the line $\delta_{B}=f\left(\delta_{A}\right)$ for $0\leq \rho_{s}\leq 1$ or the line $\delta_{B}=h\left(\delta_{A}\right)$ for $1-\leq \rho_{s}\leq 0$, and the other 1−α fraction are independently drawn from the distributions $P_{A}\left(\delta_{A}\right)$ and $P_{B}\left(\delta_{B}\right)$. Formally this can be written as

(Equation 65) $$\begin{array}{cc} P\left(\delta_{A},\delta_{B}\right) & =\left(1-\alpha \right)P_{A}\left(\delta_{A}\right)P_{B}\left(\delta_{B}\right)\\ & +\alpha \left\{ \begin{array}{ll} \delta \left(V_{A}\left(\delta_{A}\right)-V_{B}\left(\delta_{B}\right)\right)P_{A}\left(\delta_{A}\right)P_{B}\left(\delta_{B}\right) & \text{if}\;0\leq \rho_{s}\leq 1\\ \delta \left(V_{A}\left(\delta_{A}\right)+V_{B}\left(\delta_{B}\right)-1\right)P_{A}\left(\delta_{A}\right)P_{B}\left(\delta_{B}\right) & \text{if}\;-1\leq \rho_{s}\leq 0 \end{array}\right. \end{array}$$

Following the same derivation as in the section “There is a joint survival times distribution for which $\alpha \left(\rho_{s}\right)=\left|\rho_{s}\right|$” it can be shown that for the distribution given in Equation 65, the dCDA model is exact and corresponds to $\alpha \left(\rho_{s}\right)=\left|\rho_{s}\right|$. Therefore we will use this specification of $\alpha \left(\rho_{s}\right)$ in the interpolation between the cases $\rho_{s}=$-1, 0 and 1 for the dCDA model.

##### The dCDA model

We saw in an earlier section that the dCDA framework accommodates a spectrum of models that goes from the Bliss independence model at $\rho_{s}=0$ to the Higher Single Agent model at $\rho_{s}=1$ modified to accommodates for the case of sham combinations. We also noted that at $\rho_{s}=1$ the Dose Equivalence Principle appears naturally as the relation between the lethal doses. The model is sham combination compliant because when the two drugs A and B are the same, $\rho_{s}=1$, and in this case the model reduces $V_{AB}\left(D_{A},D_{B}\right)=V_{A}\left(D_{A}+D_{B}\right)$ as required. For the specification of a joint distribution of lethal doses given in Equation 65 we have that $\alpha \left(\rho_{s}\right)=\left|\rho_{s}\right|$. Therefore we can write the final dCDA model to be used in the paper as:

(Equation 66) $$\begin{array}{cc} V_{AB}\left(D_{A},D_{B},\rho_{s}\right) & =\left(1-\left|\rho_{s}\right|\right)V_{B}\left(D_{B}\right)V_{A}\left(D_{A}\right)\\ & +\left|\rho_{s}\right|\left\{ \begin{array}{ll} \min\left(V_{A}\left(D_{A}+g\left(D_{B}\right)\right),V_{B}\left(D_{B}+f\left(D_{A}\right)\right)\right) & \text{if}\;0\leq \rho_{s}\leq 1\\ \max\left(0,V_{A}\left(D_{A}\right)+V_{B}\left(D_{B}\right)-1\right) & \text{if}\;-1\leq \rho_{s}\leq 0 \end{array}\right. \end{array}$$

##### Parameterization of the viabilities using Hill curves

We can parameterize the dose-viability curve for a generic drug A $\left(V_{A}\left(D_{A}\right)\right)$ by a Hill curve with free parameters $n_{A}$ and $k_{A}$. The generic Hill curve equation often used in modeling dose response curves for drug X (X = A or B) at dose $D_{X}$ is $V_{X}\left(D_{X}\right)=\frac{1}{1+{\left(\frac{D_{X}}{k_{X}}\right)}^{n_{X}}}$ where $k_{X}$ is the dose at 50% effect and $n_{X}$ controls the steepness of the curve.

To estimate $k_{A}$, we use the experimental doses and their respective viabilities and linearly interpolate between the experimental values to obtain more data points, then regress viabilities on doses to arrive at the estimated slope and intercept. The slope and intercept of this line, $s_{A}$ and $i_{A}$ are used to compute the EC50 or $k_{A}$ value. Note that for ease of computation viabilities are transformed from 0-1 to 0-100. Therefore, we estimate $k_{A}$ by $\frac{50-i_{A}}{s_{A}}$. Then, using the functional form of the Hill curve given by the expression above for $V_{A}\left(D_{A}\right)$, we numerically estimate $n_{A}$. We slowly increase the guessed value of $n_{A}$, compute the resulting Hill curve and find the root mean square error (RMSE) at the points for which we have experimental data. The $n_{A}$ value with lowest RMSE is chosen. The same procedure can be done for $k_{B}$ and $n_{B}$. Under these parameterizations, some of the functions that we described above can be explicitly written. For example the functions for equivalent doses $f\left(D_{A}\right)$ and $g\left(D_{B}\right)$ are

(Equation 67) $$f\left(D_{A}\right)=k_{B}{\left(\frac{D_{A}}{k_{A}}\right)}^{\frac{n_{A}}{n_{B}}}$$

(Equation 68) $$g\left(D_{B}\right)=k_{A}{\left(\frac{D_{B}}{k_{B}}\right)}^{\frac{n_{B}}{n_{A}}}$$

##### Fitting the dCDA model to the data

The optimal estimate for the observed combination viability under the dCDA model can be found by estimating the parameter $\rho_{s}$, which is constrained between [−1,1], that best fits the experimental data. We first estimate the Hill curve parameters for each of the two individual drugs as described in the section above. Next, we nominate 200 equally spaced candidate values of $\rho_{s}$ on the interval [−1,1] and for each candidate $\rho_{s}$ we compute its predicted combination viability curve using Equation 66. From these 200 possible combination viability curves, we choose the $\rho_{s}$ corresponding to the curve that minimizes the RMSE between the esitmated and observed combination viabilities.

Now, we have an initial estimate for $\rho_{s}$ for a given combination using all the data points, but we have yet to account for possible outliers in the experimental data. We must find and remove outliers from the data and then re-estimate $\rho_{s}$. We first perform a linear regression between the observed viabilities and the initial combination estimates. Then, we computed the externally studentized residuals which should come from a t-distribution with n−3 (because only 1 covariate) degrees of freedom where n is the number of points in the data set. Outliers were defined as points with absolute value greater than a predetermined cutoff $\left(1-\frac{0.05}{2n}\right)$ based on n, the number of total points. There was less than 1 outlier on average found per combination (0.615 outliers/combination). Once we remove the outliers from the data, we estimate $\rho_{s}$ using the exact same procedure as in the preceding paragraph. The correlation estimate did not change for 84% of the combinations after removing outliers which suggests that the dCDA model is robust. The complete data regarding number of outliers, and before and after removing outliers optimal correlation estimate can be found in Table S2. We use the outlier removed correlation estimates for further analysis.

##### Quantification of combinations not accounted for by the dCDA model

Excess over Bliss (EOB) is a commonly used metric for assessing synergistic and antagonistic drug combination effects. It is calculated as $EOB=V_{A}\left(D_{A}\right)V_{B}\left(D_{B}\right)-V_{AB}\left(D_{A},D_{B}\right)$. By computing EOB, the assumption is that Bliss independence represents the null model. We tested the assumption that Bliss independence is a valid null model by comparing the observed combination viabilities to the Bliss independence viabilities ($\rho_{s}=0$ in the dCDA model) and computing a p-value based on a two-sample paired t-test. A p-value > 0.01 suggests that the EOB condition adequately provides a measure of synergy/antagonism in the given combination.

We introduce a more general form of deviation from the dCDA null model: the Excess over CDA (EOCDA) - which, for different values of $\rho_{s}$, ranges from Bliss independence to the sham compliant HSA model depending on the optimal $\rho_{s}$ value. EOCDA is measured by the difference between the estimated combination viabilities under dCDA and the observed combination viability. A non-zero EOCDA indicates some sort of interaction between the drugs: EOCDA greater than 0 suggests synergy and an EOCDA less than 0 suggests antagonism. The magnitude of the difference indicates the deviation from independent drug action.

Once an optimal estimate for the combination viabilities has been found under dCDA, goodness of fit must be assessed. A two-sample paired t-test was used to compute a p-value indicating evidence against the null hypothesis that the CDA estimate describes the observed combination. For a single hypothesis test, we set the p-value threshold at 0.05, a standard level. However, we test multiple hypotheses. We adjust for this by using a Benjamini-Hochberg correction wherein the actual threshold is the individual threshold over the number of trials. This value of 0.0019 is the threshold for rejecting or failing to reject the null model. A p-value less than this threshold suggests that the null hypothesis should be rejected and that there exists a non-dCDA process that better describes the combination. Results for the dCDA model on the tested combinations can be found in Table S2.

##### Identifying locally possibly synergistic or antagonistic dosages in a globally independent joint action drug combination2

We want to identify which, if any, points in a given dCDA independent-joint-action drug combination are possibly acting non-independently (i.e., possibly acting synergistically or antagonistically). Here, a point refers to a given dose of each monotherapy and its associated optimal dCDA estimated viability and the observed viability. We can plot the estimated and observed viability (Figures 2D and 2F) and visually, those points that are far from the identity are candidates to be locally synergistic or antagonistic dosages. We will formalize this notion now.

First, let us compute the residuals across all points (estimate - observed). We then assume that this distribution is asymptotically normal and estimate the mean $\left(\hat{\mu }\right)$ and variance $\left(\hat{\sigma^{2}}\right)$ of its corresponding normal distribution using the MLE estimate for these parameters.

We remove a given point i from the set. Then, we re-estimate the optimal ρ using the dCDA model and arrive at a new optimal estimate for the viabilities. Next, using the newly optimal ρ, we compute the estimated value at the held out point and the residual at this point $\left(r_{i}\right)$. Finally, we compute the z-score of this point $\left(\frac{\hat{\mu }-r_{i}}{\hat{\sigma }}\right)$ where $\hat{\sigma }$ is the square root of $\hat{\sigma^{2}}$. Then, we compute the two-sided p-value using this z-score in reference to a standard normal distribution. If the p-value is greater than 0.01, then we fail to reject the null hypothesis that point i is consistent with the dCDA model.

### Quantification and statistical analysis

R version 4.1.2 was used for all analysis. The statistical measure for comparing the tCDA model fit to the measured survival probabilities is described in the STAR Methods Section “Fitting the tCDA model to the data”. For dCDA, the procedure for assessing goodness of fit of the model estimate is described in the STAR Methods Section “Quantification of combinations not accounted for by the dCDA model”. And, the statistical testing framework to identify locally non-dCDA doses is described in the STAR Methods Section “Identifying locally possibly synergistic or antagonistic dosages in a globally independent joint action drug combinations”.

## Supplemental information

Document S1. Figures S1–S18

File S1. Clinical Trial PFS Data, related to Figure 1 Aggregated raw clinical trial progression-free survival data.

File S2. Cell Line Combination Experiment Data, related to Figure 2 Raw data for the experimentally tested combinations.

Table S1. tCDA Model Results, related to Figure 1 Clinical trial results with Trial IDs and corresponding results for each clinical trial combination.

Table S2. dCDA Model Results, related to Figure 2 Experimentally tested cell line combination results with experiment IDs.

Table S3. Clinical Trial Data Sources, related to Figure 1 Guide describing the sources from which the clinical trial data was assembled.

## References

[1] Kurth A.E., Celum C., Baeten J.M., Vermund S.H., Wasserheit J.N. (2011). Combination HIV Prevention: Significance, Challenges, and Opportunities. Curr. HIV AIDS Rep..

[2] Frei E., Freireich E.J., Gehan E., Pinkel D., Holland J.F., Selawry O., Haurani F., Spurr C.L., Hayes D.M., James G.W. (1961). Studies of Sequential and Combination Antimetabolite Therapy in Acute Leukemia: 6-Mercaptopurine and Methotrexate. Blood.

[3] Freireich E.J., Gehan E., Frei E., Schroeder L.R., Wolman I.J., Anbari R., Burgert E.O., Mills S.D., Pinkel D., Selawry O.S. (1963). The Effect of 6-Mercaptopurine on the Duration of Steroid-induced Remissions in Acute Leukemia: A Model for Evaluation of Other Potentially Useful Therapy. Blood.

[4] Mokhtari R.B., Homayouni T.S., Baluch N., Morgatskaya E., Kumar S., Das B., Yeger H. (2017). Combination therapy in combating cancer. Oncotarget.

[5] Frei E., Karon M., Levin R.H., Freireich E.J., Taylor R.J., Hananian J., Selawry O., Holland J.F., Hoogstraten B., Wolman I.J. (1965). The Effectiveness of Combinations of Antileukemic Agents in Inducing and Maintaining Remission in Children with Acute Leukemia. Blood.

[6] Fitzgerald J.B., Schoerberl B., Nielsen U.B., Sorger P.K. (2006). Systems Biology and Combination Therapy in the Quest for Clinical Efficacy. Nat. Chem. Biol..

[7] Palmer A.C., Sorger P.K. (2017). Combination Cancer Therapy Can Confer Benefit via Patient-to-Patient Variability without Drug Additivity or Synergy. Cell.

[8] Bliss C.I. (1939). The toxicity of poisons applied jointly 1. Ann. Appl. Biol..

[9] Plana D., Palmer A.C., Sorger P.K. (2022). Independent Drug Action in Combination Therapy: Implications for Precision Oncology. Cancer Discov..

[10] Schmidt E.V., Sun L.Z., Palmer A.C., Chen C. (2023). Rationales for Combining Therapies to Treat Cancer: Independent Action, Response Correlation, and Collateral Sensitivity Versus Synergy. Annu. Rev. Cancer Biol..

[11] Chen C., Liu F., Ren Y., Suttner L., Sun Z., Shentu Y., Schmidt E.V. (2020). Independent drug action and its statistical implications for development of combination therapies. Contemp. Clin. Trials.

[12] Geeleher P., Cox N.J., Huang R.S. (2016). Cancer biomarker discovery is improved by accounting for variability in general levels of drug sensitivity in pre-clinical models. Genome Biol..

[13] Bliss C.I. (1956). The Calculation of Microbial Assays. Bacteriol Rev..

[14] Schmidt E.V., Chisamore M.J., Chaney M.F., Maradeo M.E., Anderson J., Baltus G.A., Pinheiro E.M., Uebele. V.N. (2019). Assessment of Clinical Activity of PD-1 Checkpoint Inhibitor Combination Therapies Reported in Clinical Trials. JAMA Netw. Open.

[15] Gao H., Korn J.M., Ferretti S., Monahan J.E., Wang Y., Singh M., Zhang C., Schnell C., Yang G., Zhang Y. (2015). High-throughput screening using patient-derived tumor xenografts to predict clinical trial drug response. Nat. Med..

[16] Loewe S. (1953). The Problem of Synergism and Antagonism of Combined Drugs. Arzneimittelforschung.

[17] Tang J., Wennerberg K., Aittokallio T. (2015). What is Synergy? The Saariselkä agreement revisted. Front. Pharmacol..

[18] Lugowska I., Kosela-Patercyzk H., Kozak K., Rutkowski P. (2015). Trametinib: a MEK Inhibitor for Management of Metastatic Melanoma. OncoTargets Ther..

[19] Bowyer S., Lee R., Fusi A., Lorigan P. (2015). Dabrafenib and Its Use in the Treatment of Metastatic Melanoma. Melanoma Manag..

[20] Santos L.C., Vogel R., Chipuk J.E., Birtwistle M.R., Stolovitzky G., Meyer P. (2019). Mitochondrial origins of fractional control in regulated cell death. Nat. Commun..

[21] Gottardis M.M., Jordan V.C. (1987). Antitumor Actions of Keoxifene and Tamoxifen in the Nnitrosomethylurea- induced Rat Mammary Carcinoma Model. Cancer Res..

[22] Hsieh Y.-Y., Chou C.-J., Lo H.-L., Yang P.-M. (2016). Repositioning of a Cyclin-Dependent Kinase Inhibitor GW8510 as a Ribonucleotide Reductase M2 Inhibitor to Treat Human Colorectal Cancer. Cell Death Discov..

[23] Diaz J.E., Ahsen M.E., Schaffter T., Chen X., Realubit R.B., Karan C., Califano A., Losic B., Stolovitzky G. (2020). The Transcriptomic Response of Cells to a Drug Combination is More Than The Sum of the Responses to the Monotherapies. Elife.

[24] Slamon D.J., Leyland-Jones B., Shak S., Fuchs H., Paton V., Bajamonde A., Fleming T., Eiermann W., Wolter J., Pegram M. (2001). Use of chemotherapy plus a monoclonal antibody against HER2 for metastatic breast cancer that overexpresses HER2. N. Engl. J. Med..

[25] Alexander L., Huang R.S. (2020). Computationally predicting clinical drug combination efficacy with cancer cell line screens and independent drug action. Nat. Commun..

[26] Greco W.R., Bravo G., Parsons J.C. (1995). The search for synergy: a critical review from a response surface perspective. Pharmacol. Rev..

[27] Loewe S. (1953). The problem of synergism and antagonism of combined drugs. Arzneimittelforschung.

[28] Chou T.-C., Talalay P. (1984). Quantitative analysis of dose-effect relationships: the combined effects of multiple drugs or enzyme inhibitors. Adv. Enzym. Regul..

[29] Grabovsky Y., Tallarida R.J. (2004). Isobolographic analysis for combinations of a full and partial agonist: curved isoboles. J. Pharmacol. Exp. Ther..

[30] Foucquier J., Guedj M. (2015). Analysis of drug combinations: current methodological landscape. Pharmacol. Res. Perspect..

[31] Meyer C.T., Wooten D.J., Paudel B.B., Bauer J., Hardeman K.N., Westover D., Lovly C.M., Harris L.A., Tyson D.R., Quaranta V. (2019). Quantifying Drug Combination Synergy along Potency and Efficacy Axes. Cell Syst..

[32] Wooten D.J., Meyer C.T., Quaranta V., Lopez C. (2019). A consensus framework unifies multi-drug synergy metrics. bioRxiv.

[33] Berenbaum M.C. (1989). What is synergy?. Pharmacol. Rev..

