# Supplementary material for: Modeling combination therapies in patient cohorts and cell cultures using correlated drug action
Source: iScience. 2024 Jan 15;27(3):108905. doi: 10.1016/j.isci.2024.108905 (PMC10882105; doi:10.1016/j.isci.2024.108905)
Supplement: Document S1. Figures S1–S18 [file mmc1.pdf]

iScience, Volume *27*

Supplemental information

Modeling combination therapies in patient cohorts and cell cultures using correlated drug action

Adith S. Arun, Sung-Cheol Kim, Mehmet Eren Ahsen, and Gustavo Stolovitzky

Supplementary Information

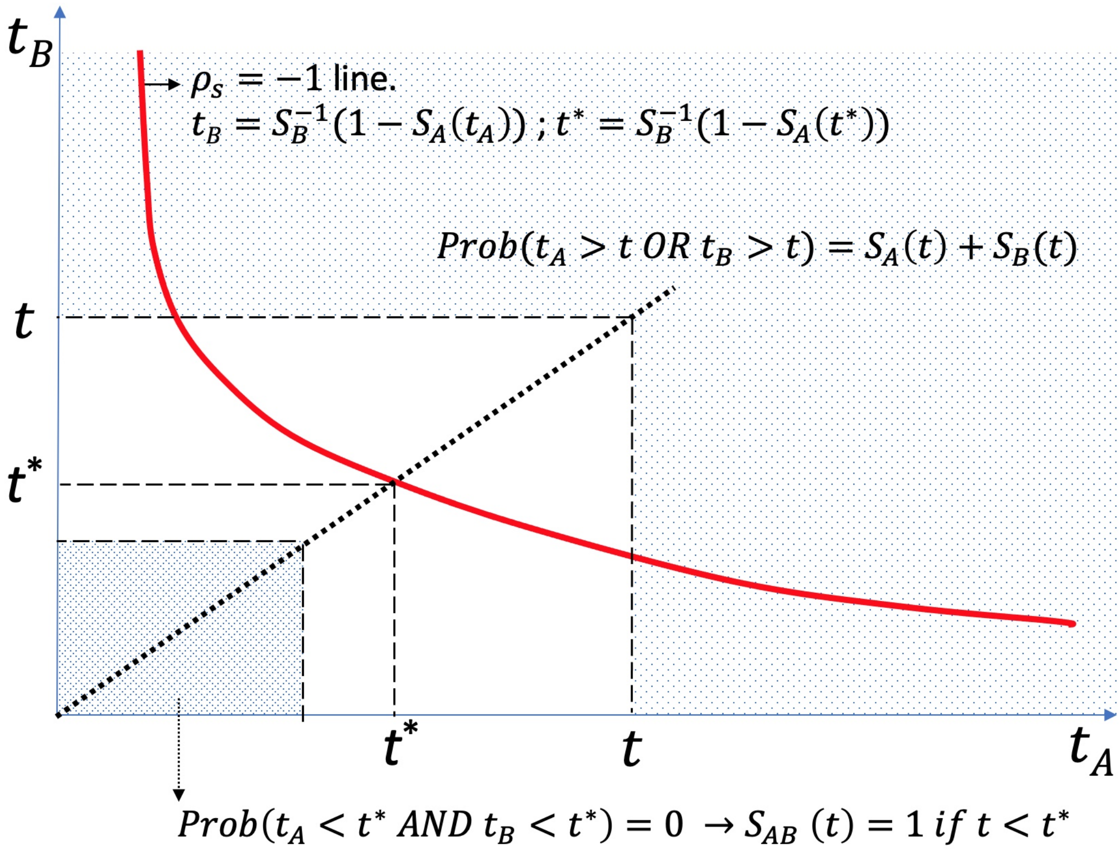

Figure S1: Diagram of survival times for the case when $\rho_s = -1$ in the temporal CDA setup. Related to Figure 1

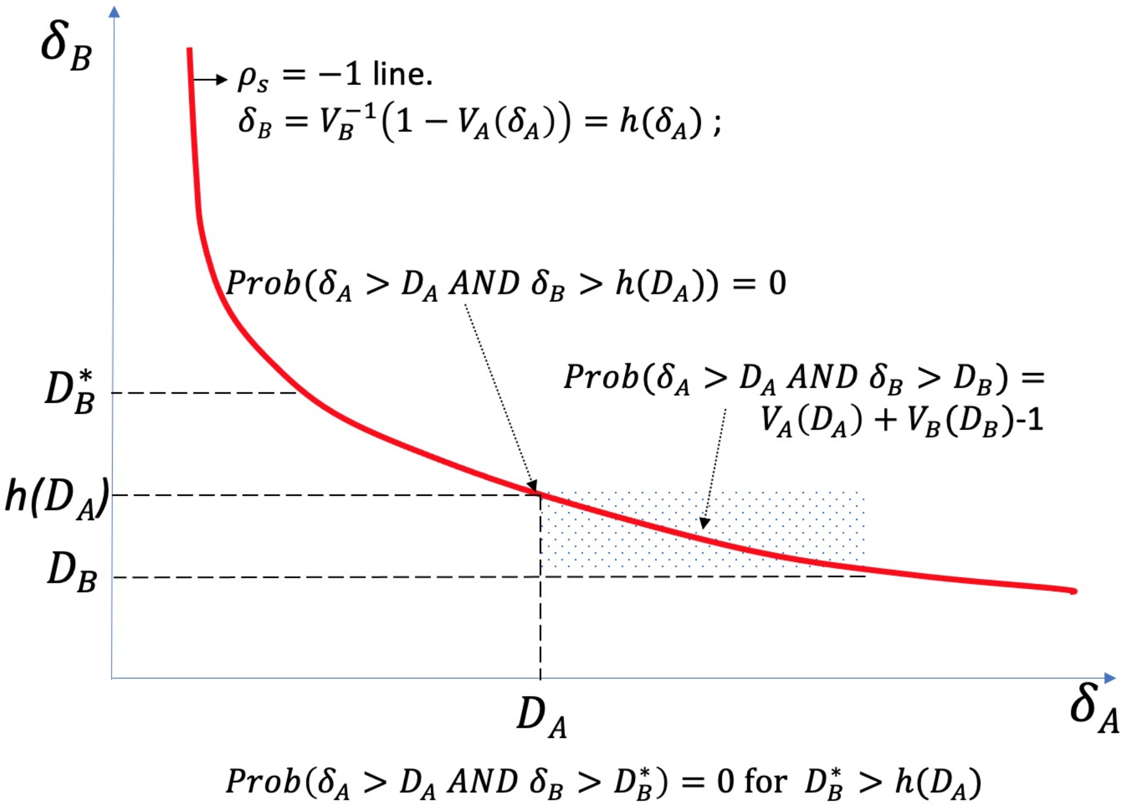

Figure S2: Diagram of minimal killing doses for cells in culture for the case when $\rho_s = -1$ in the dose CDA setup. Related to Figure 2.

Supplemental Figures

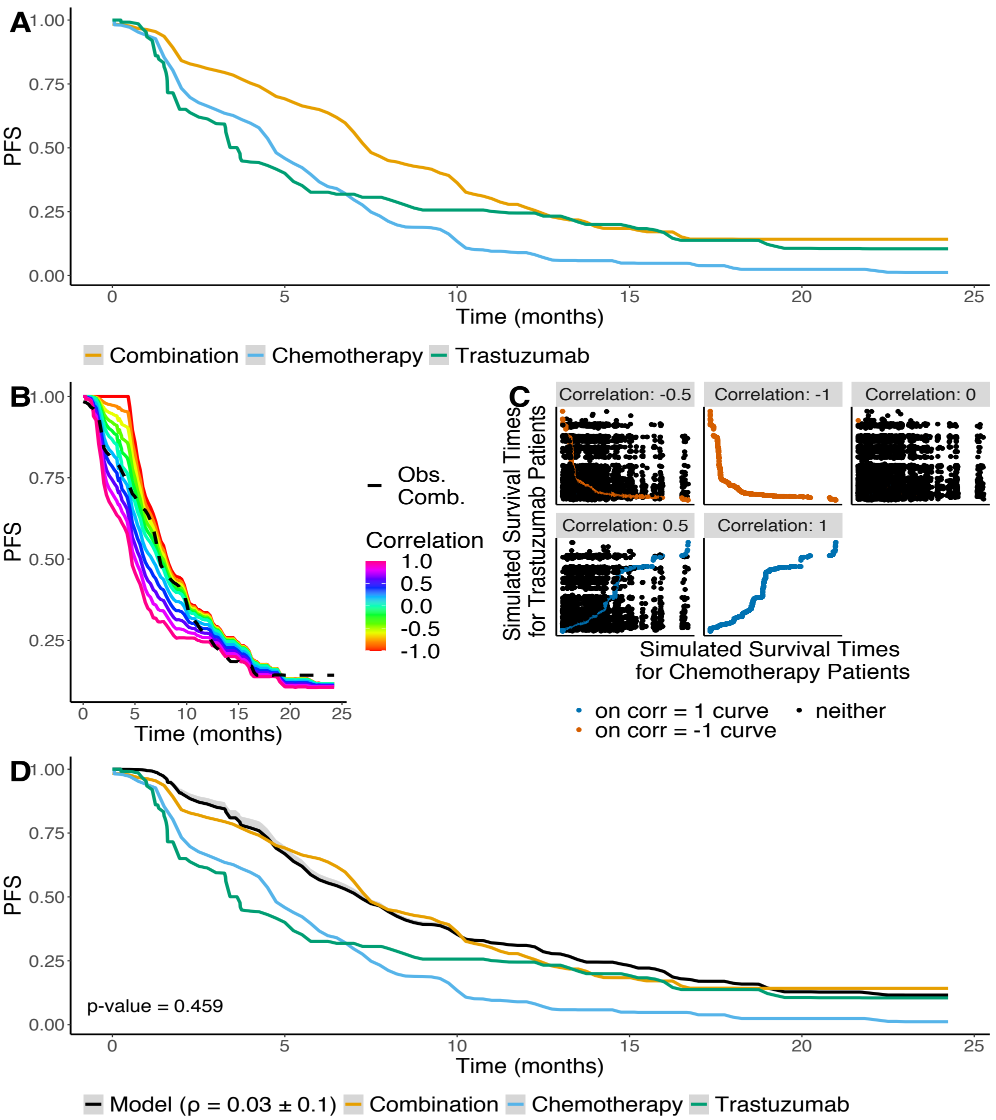

Figure S3: Correlated Drug Action explains the benefit of Trastuzumab and Chemotherapy in Metastatic HER-2 Overexpressing Breast Cancer. **A)** Progression Free Survival (PFS) as a function of time for either individual therapy option (Trastuzumab - green, Chemotherapy - blue) and their combination (yellow) in patients with HER-2 overexpressing breast cancer. **B)** Range of possible survival curves for the combination under tCDA. The observed combination (black) falls within the lines of the predicted field. **C)** For a given Spearman's correlation, each point represents a pair of possible PFS times associated with each simulated patient. The parallel maximum, maximum survival time between the coordinates of each point, of this paired vector of PFS times defines the combination PFS curve shown in **B**. The absolute value of the Spearman's correlation corresponds to the fraction of points that lie on the correlation -1 or 1 curves (Fig. S7). **D)** Estimate of the combination under the tCDA model (black) and in grey are the PFS curves for the 95% confidence interval of ρ_s (0.03 ± 0.1, p-value $= 0.459$). This suggests that the tCDA model sufficiently describes the effect of the combination. Related to Figure 1.

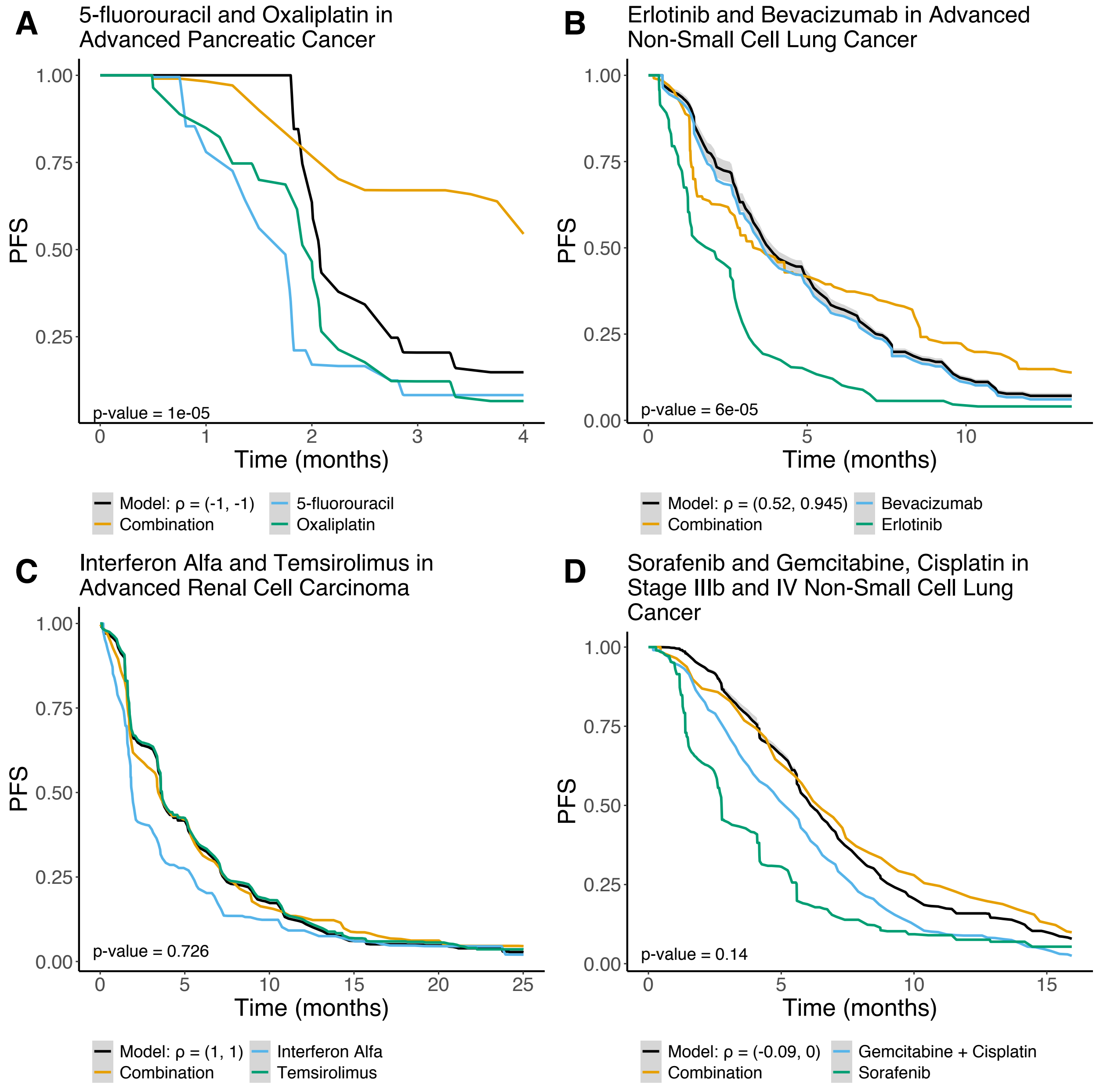

Figure S4: tCDA model results. **A)** Combination of 5-fluorouracil and Oxaliplatin in advanced pancreatic cancer. **B)** Combination of Erlotinib and Bevacizumab in advanced non-small cell lung cancer. **C)** Combination of Interferon Alfa and Temsirolimus in advanced renal cell carcinoma. Since the optimal Spearman's correlation is 1, the tCDA converges to simply following the monotherapy PFS curve with higher PFS at each time step. The model estimate (black) is equivalent to that of Temsirolimus monotherapy (green), the better performing monotherapy. **D)** Combination of Sorafenib and Gemcitabine, Cisplatin in Stage IIIb and IV Non-Small Cell Lung Cancer. **A, B, C, D)** For a given clinical trial, the estimated combination survival curve (black), associated 95% confidence interval (grey) under tCDA, p-value regarding goodness-of-fit, and optimal Spearman's correlation estimate are shown alongside the individual monotherapies and observed combination PFS curves. Related to Figure 1.

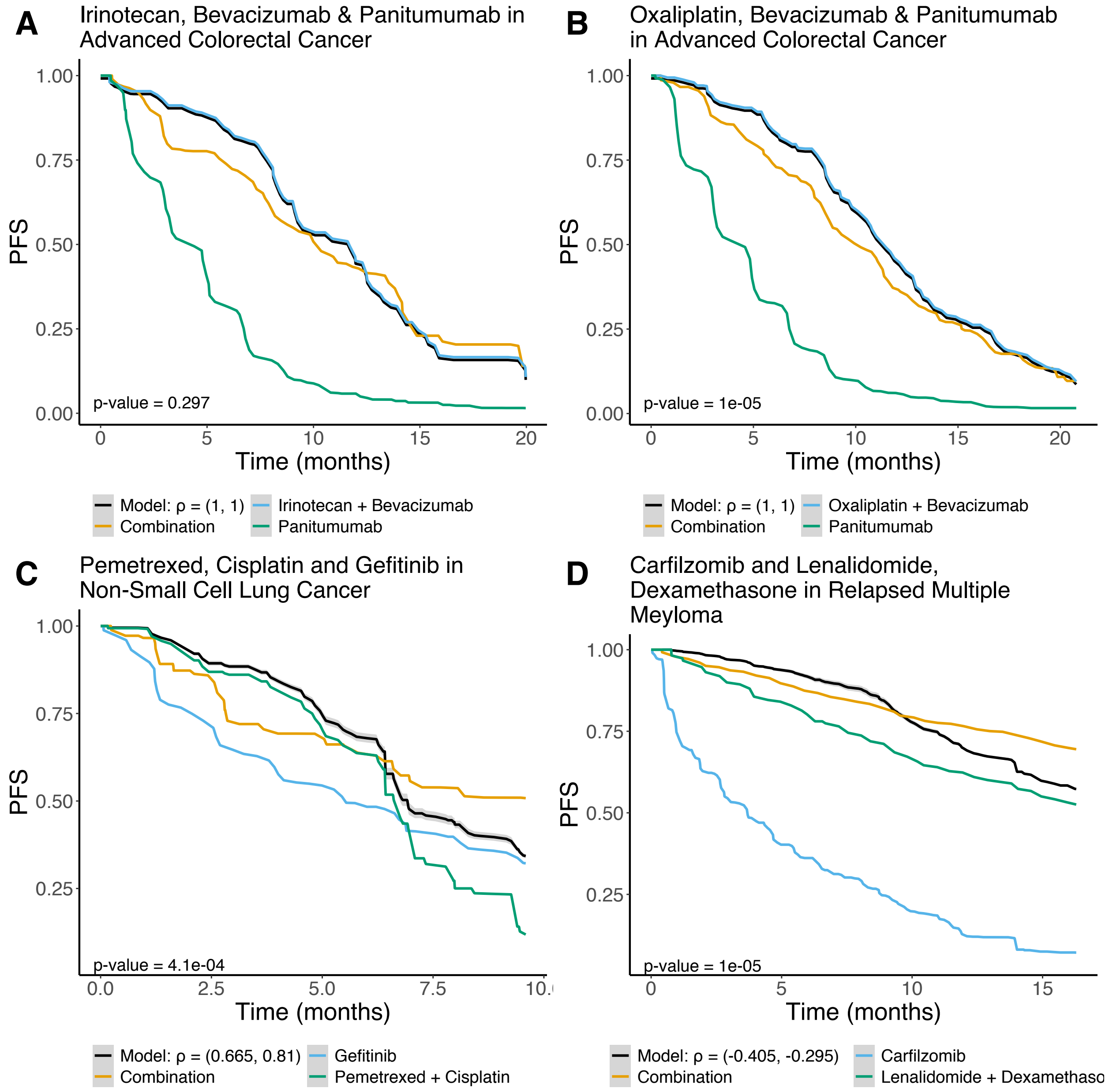

Figure S5: tCDA model results. A) Combination of Irinotecan, Bevacizumab, and Panitumumab in advanced colorectal cancer. **B)** Combination of Oxaliplatin, Bevacizumab and Pantiumumab in advanced colorectal cancer. **A,B)** Since the optimal Spearman's correlation is 1, the tCDA converges to simply following the monotherapy PFS curve with higher PFS at each time step. Therefore, the model estimate (black) is equivalent to that of the better performing monotherapy (blue). **C)** Combination of Pemetrexed, Cisplatin, and Gefitinib in non-small cell lung cancer. **D)** Combination of Carfilzomib, Lenalidomide and Dexamethasone in relapsed multiple myeloma. **A, B, C, D)** For a given clinical trial, the estimated combination survival curve (black), associated 95% confidence interval (grey) under tCDA, p-value regarding goodness-of-fit, and optimal Spearman's correlation estimate are shown alongside the individual monotherapies and observed combination PFS curves. Related to Figure 1.

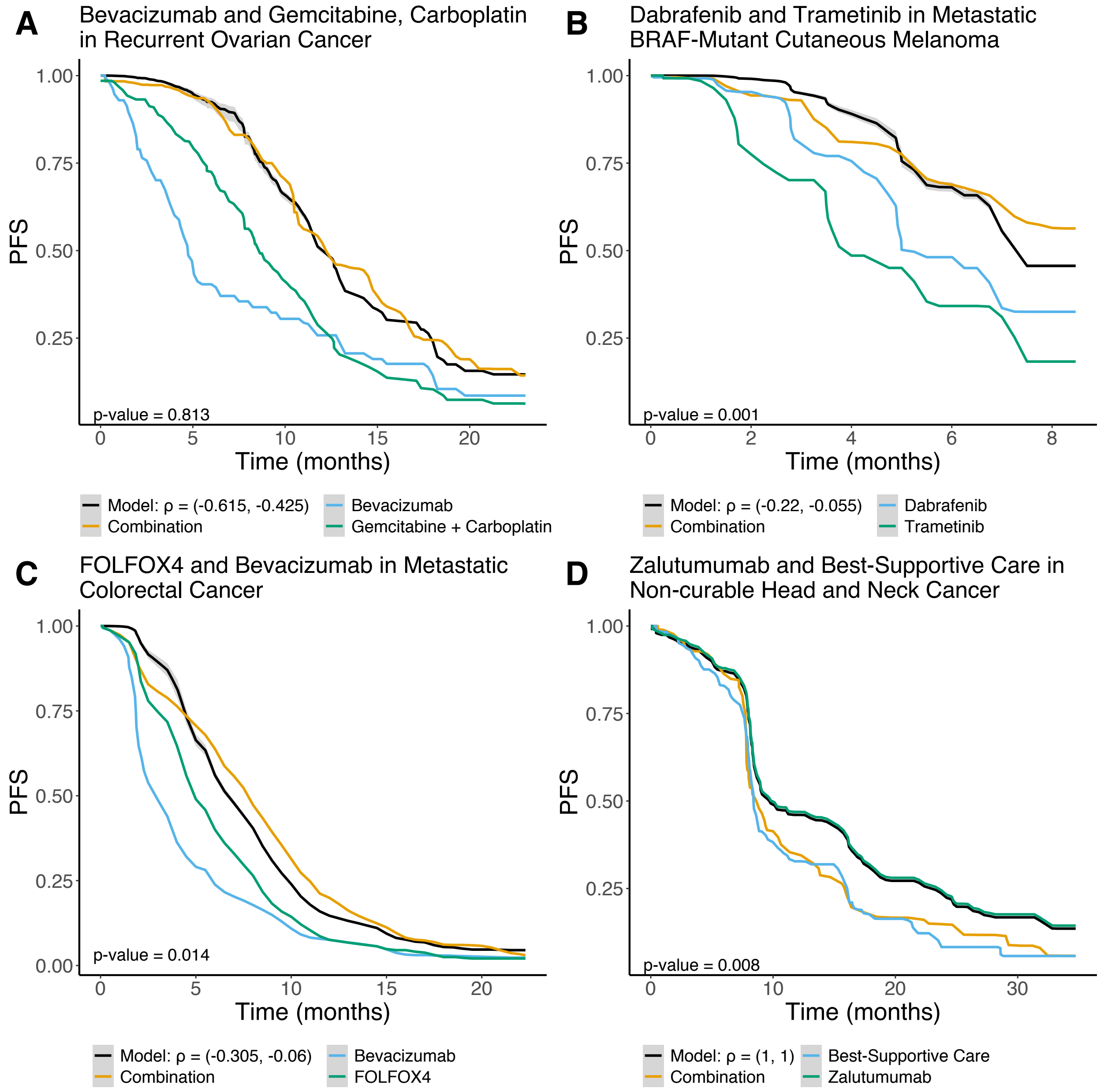

Figure S6: tCDA model results. A) Combination of Bevacizumab, Gemcitabine, and Carboplatin in recurrent ovarian cancer. **B)**Combination of Dabrafenib and Trametinib in metastatic BRAF-mutant cutaneous melanoma. **C)** Combination of FOLFOX4 and Bevacizumab in metastatic colorectal cancer. **D)** Combination of Zalutumumab and best-supportive care in non-curable head and neck cancer. Since the optimal Spearman's correlation is 1, the tCDA converges to simply following the monotherapy PFS curve with higher PFS at each time step. The model estimate (black) is equivalent to that of the Zalatumumab PFS curve (green), the better performing monotherapy. **A, B, C, D)** For a given clinical trial, the estimated combination survival curve (black), associated 95% confidence interval (grey) under tCDA, p-value regarding goodness-of-fit, and optimal Spearman's correlation estimate are shown alongside the individual monotherapies and observed combination PFS curves. Related to Figure 1.

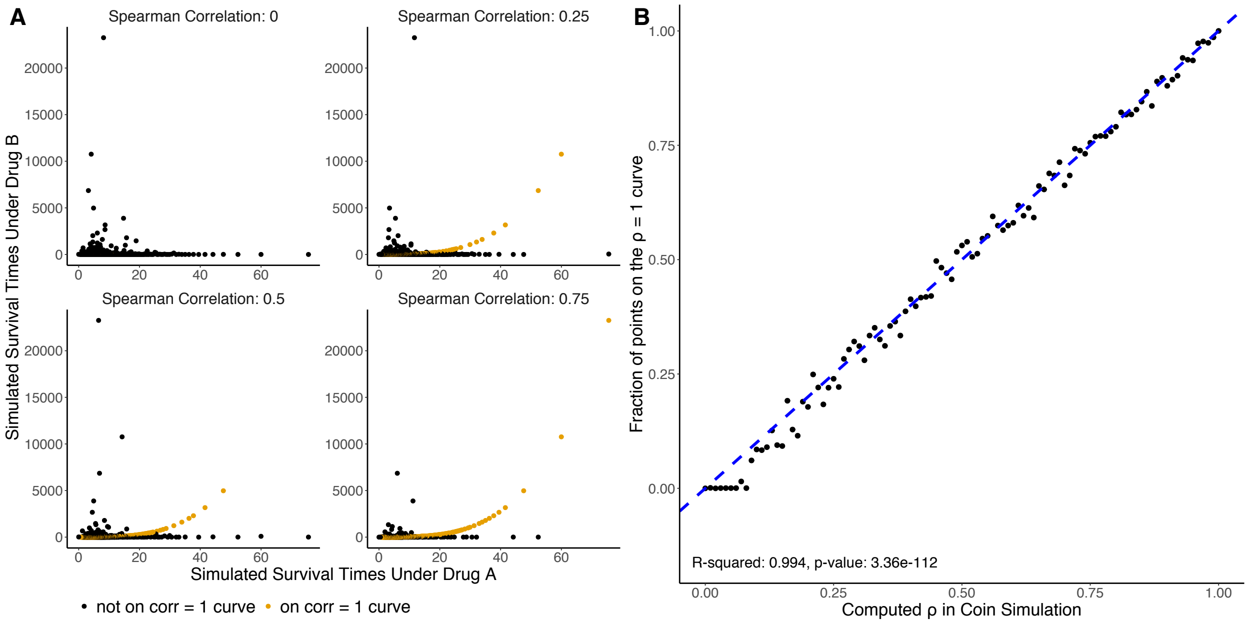

Figure S7: Rank correlation under the coin method of simulation describes the fraction of points that lie on the perfect rank correlated curve. A) Simulated data was created and the data was randomized with four distinct values of Spearman's correlation under the coin method of simulation (See Methods). The points that lie on the correlation 1 curve are colored in orange. As the correlation increases, so too does the number of points that lie on the Spearman correlation 1 curve. **B)** An alternative interpretation of the Spearman correlation in the coin method is that it represents the fraction of points that lie on the Spearman correlation equals one curve (R-squared = 0.994 , p-value = 3.4e-112). The dashed blue line is the identity. Related to Figure 1.

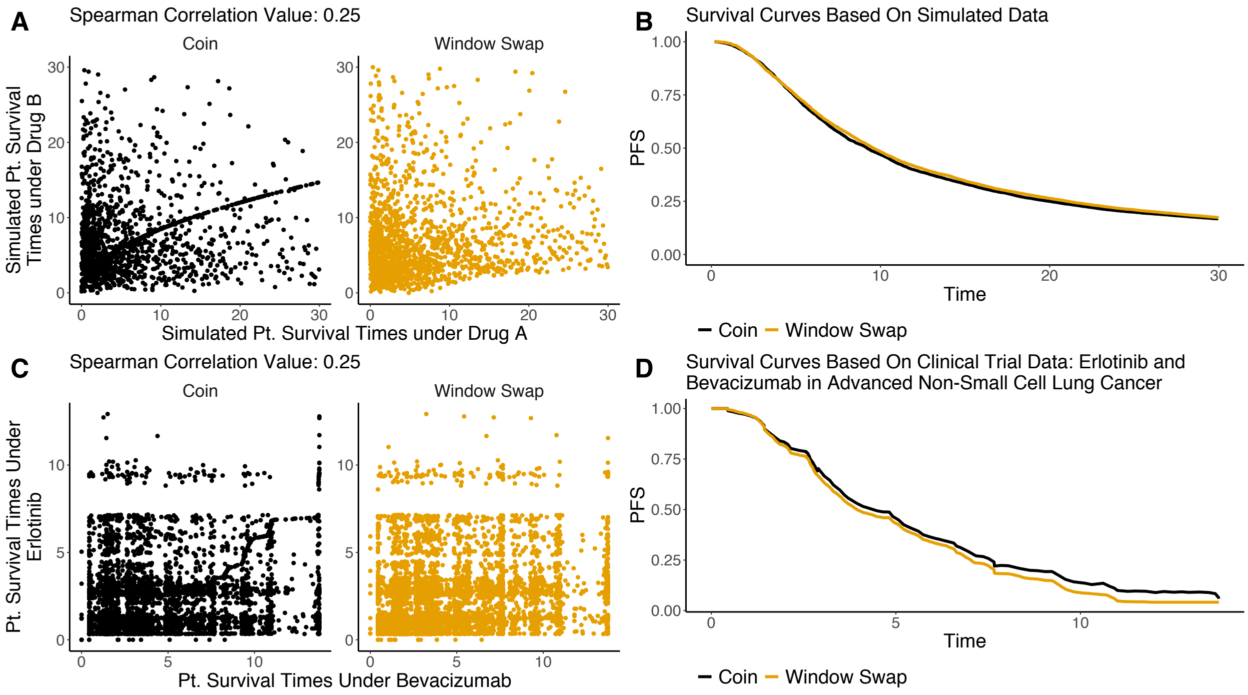

Figure S8: Differences in performance of simulation methods under synthetic and real data. **A)** Simulated data was created to create survival times under drug A and drug B. The times were randomized with Spearman correlation 0.25 under two different simulation methods - coin and window swap (see Methods) . For each simulated patient, their respective times of survival under the individual therapies are shown. **B)** The corresponding survival curves produced by both simulation methods (panel **A**) are shown and are highly concordant despite clear differences between the joint distributions shown in **A**. **C)** Data of the individual therapies from the clinical trial of Erlotinib and Bevacizumab in Advanced Non-Small Cell Lung Cancer were taken and randomized with Spearman correlation 0.25 under the coin and window swap simulation methods. The resulting survival times for a patient are represented as points in the plots. **D)** The resulting survival curves using the data from **C** are shown. The noise inherent within real data contributes to the difference between the survival curves. Related to Figure 1.

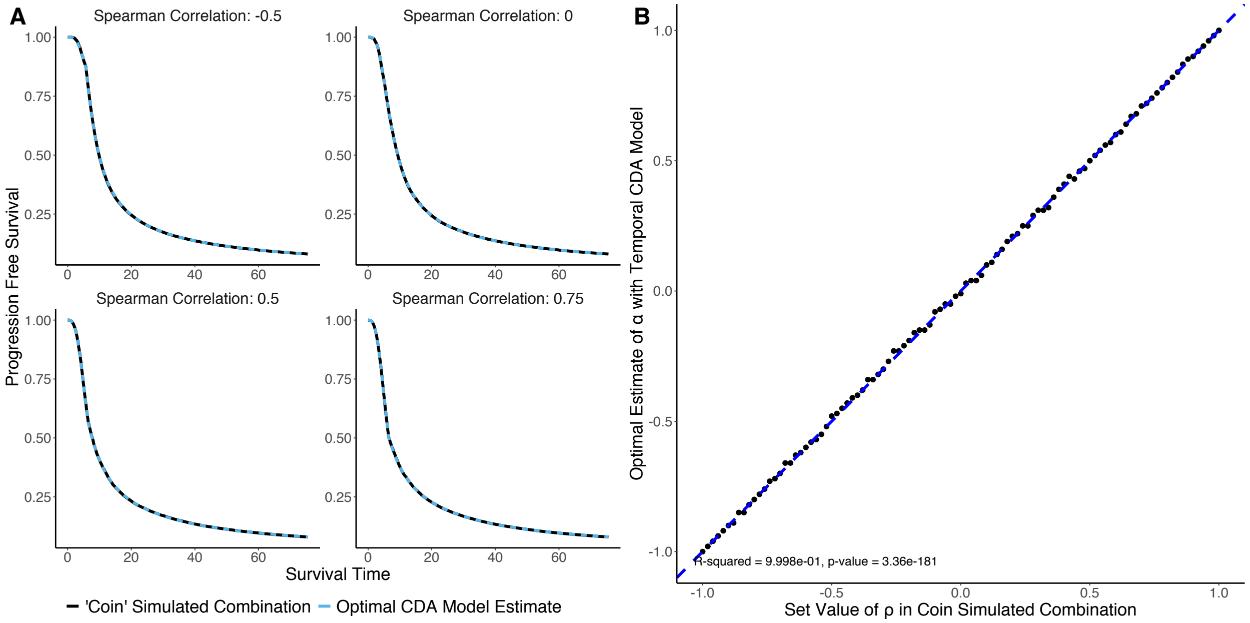

Figure S9: tCDA model is equivalent to the coin method of simulation. A) A drug combination was simulated using the coin method of randomization with specified Spearman's correlation and the tCDA model was employed to find a best estimate. The coin simulated combination and tCDA model estimates are highly concordant. **B)** Combination results were simulated with the coin method (See Methods) and estimated with the tCDA model. This was done for Spearman correlations between -1 and 1 with a step-size of 0.02. The input Spearman's correlation for the coin method and the output optimal estimate for the free parameter α in the tCDA model was plotted (R-squared = 0.9997, p-value = 4.36e-178). The dashed blue line is the identity. Related to Figure 1.

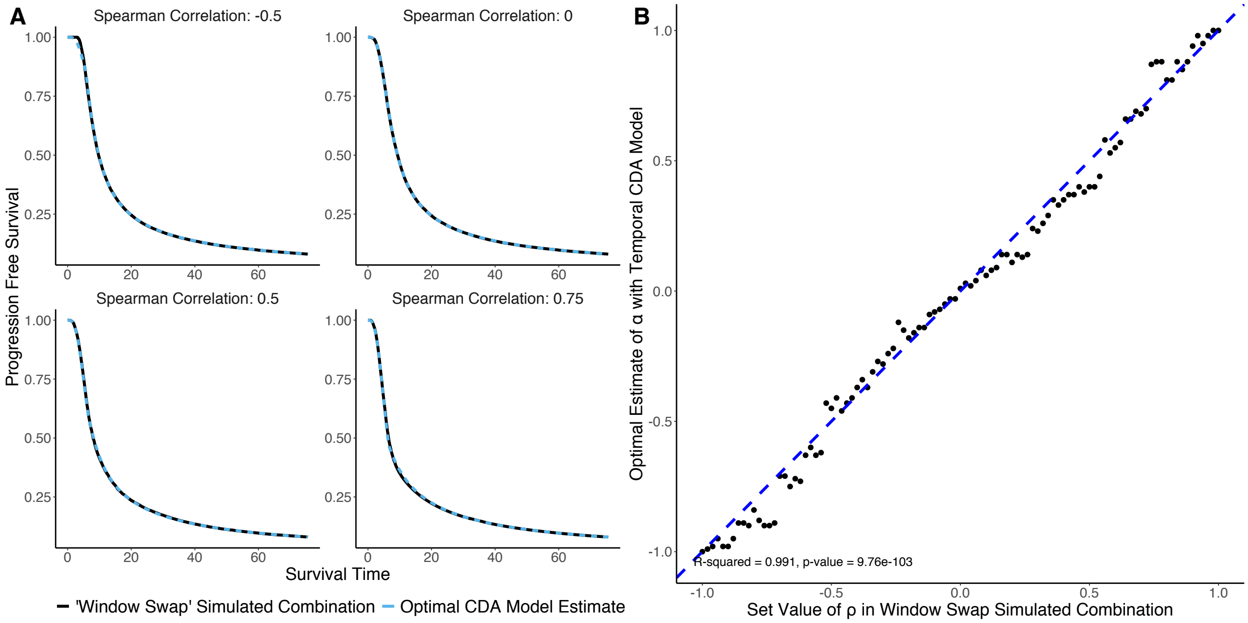

Figure S10: tCDA model captures much of the variance produced by the window swap method of simulation. **A)** A drug combination was simulated using the window swap method of randomization with specified Spearman's correlation and the tCDA model was employed to find a best estimate. **B)** Combination results were simulated with the window swap method (See Methods) and estimated with the tCDA model. This was done for Spearman correlations between -1 and 1 with a step-size of 0.02. The input Spearman's correlation for the window swap method and output optimal estimate for the free parameter α in the tCDA model were plotted (R-squared = 0.992, p-value = 8.69e-106). The dashed blue line is the identity. Related to Figure 1.

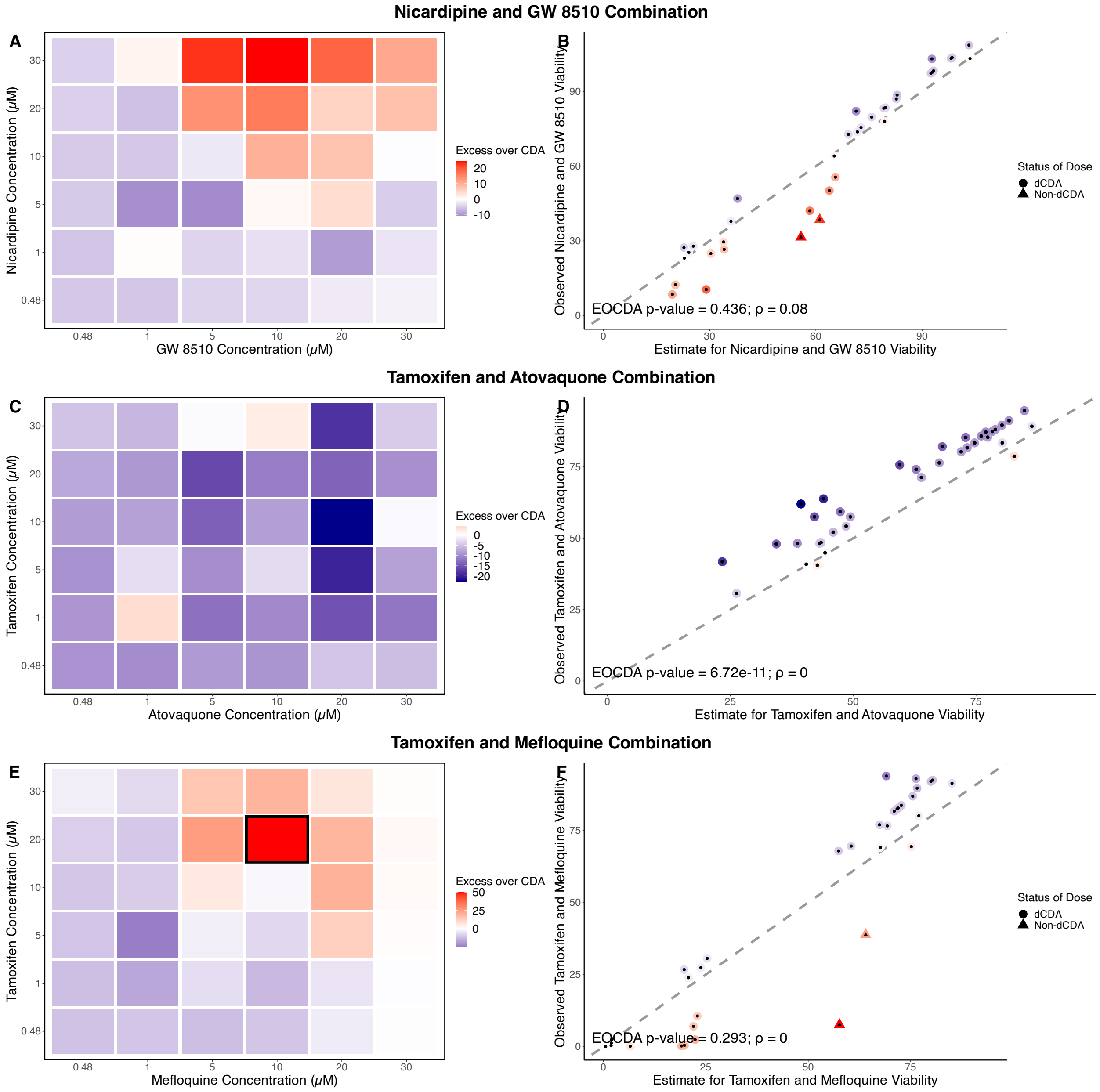

Figure S11: dCDA model results. Each row corresponds to a given combination. **A, B)** Nicardipine and GW 8510 combination in MCF7 cells collected after 24 hours. **C, D)** Tamoxifen and Atovaquone combination in MCF7 cells collected after 24 hours. **E, F)** Tamoxifen and Mefloquine combination in MCF7 cells collected after 24 hours. **A,C,E)** Heatmap of excess over CDA is shown with outlier cells bordered in black. **B, D, F)** Comparison of combination estimates and observed viabilities along with goodness-of-fit (GoF) p-value and corresponding optimal Spearman correlation's estimate. Points are colored with the same scale as its corresponding EOCDA matrix. If the GoF p-value > 0.01 for the overall combination, then each point (i.e., dose) is classified as following the dCDA model or not (i.e., likely synergistic or antagonistic behavior). Related to Figure 2.

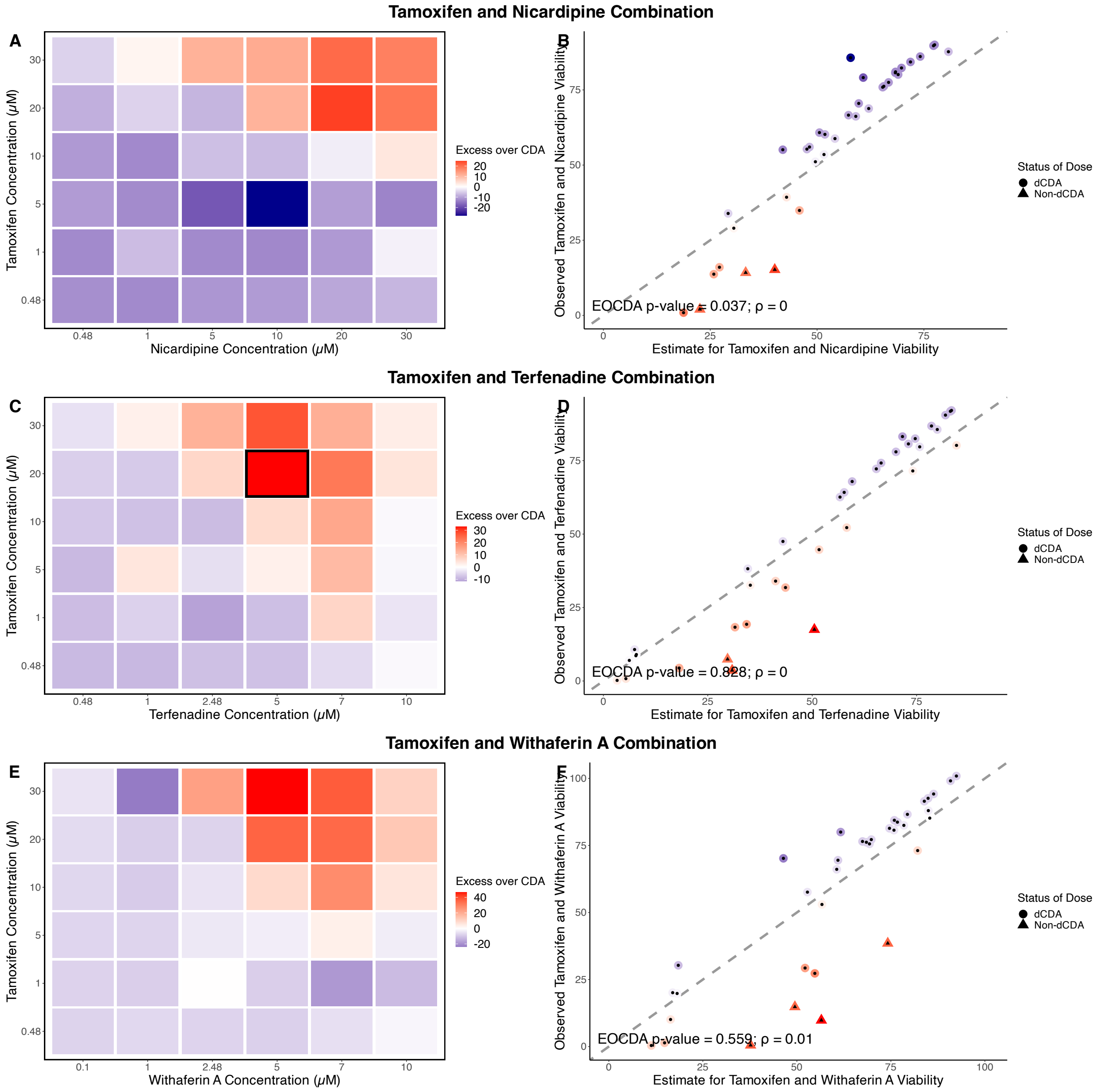

Figure S12: dCDA model results. Each row corresponds to a given combination. **A, B)** Tamoxifen and Nicardipine in MCF7 cells. **C, D)** Tamoxifen and Terfenadine combination in MCF7 cells collected after 24 hours. **E, F)** Tamoxifen and Withaferin A in MCF7 cells collected after 24 hours. **A, C, E)** Heatmap of excess over CDA is shown with outlier cells bordered in black. **B, D, F)** Comparison of combination estimates and observed viabilities along with goodness-of-fit (GoF) p-value and corresponding optimal Spearman correlation's estimate. Points are colored with the same scale as its corresponding EOCDA matrix. If the GoF p-value > 0.01 for the overall combination, then each point (i.e., dose) is classified as following the dCDA model or not (i.e., likely synergistic or antagonistic behavior). Related to Figure 2.

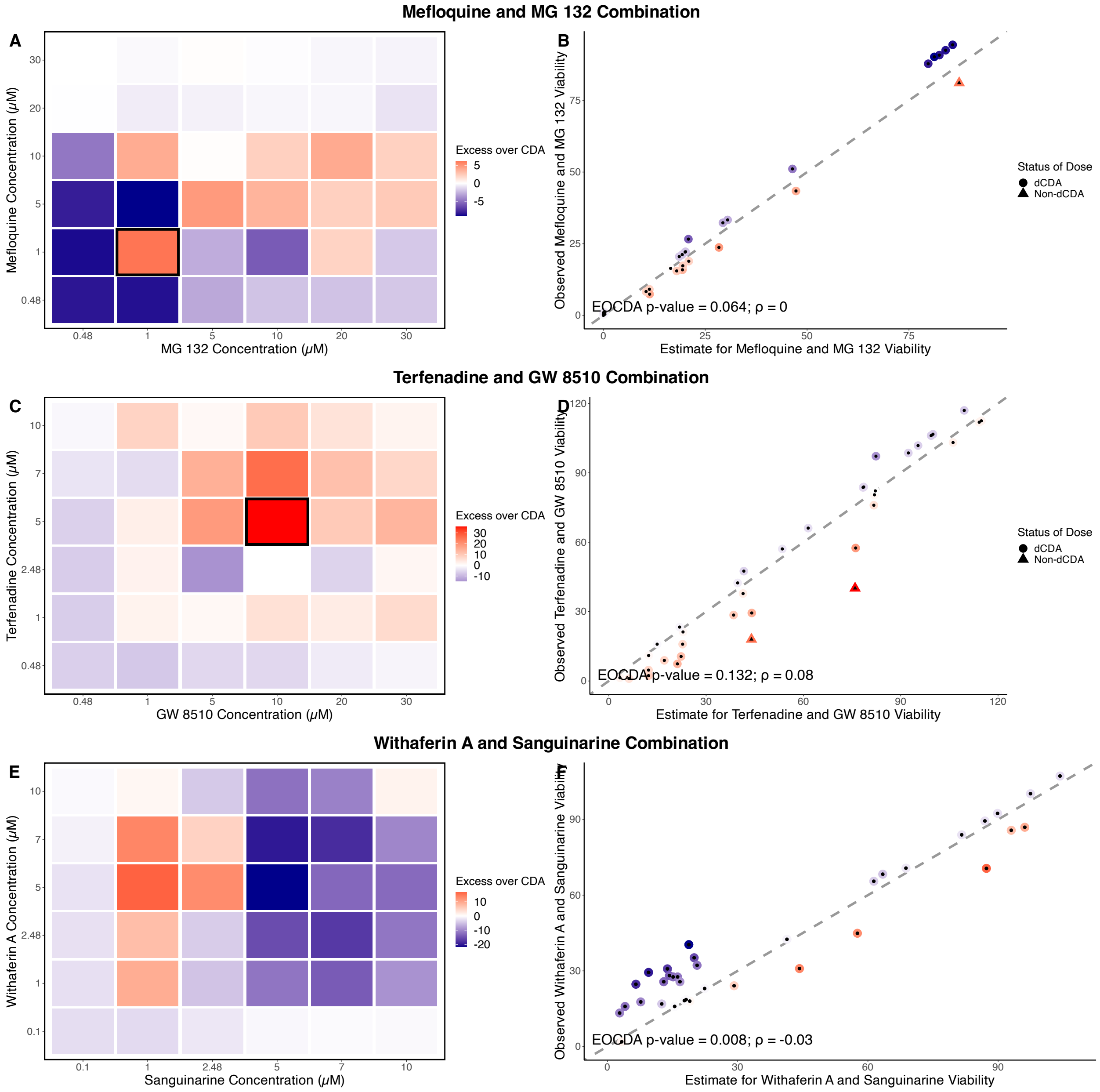

Figure S13: dCDA model results. Each row corresponds to a given combination. **A, B)** Tamoxifen and MG 132 in MCF7 cells. **C, D)** GW 8510 and Terfenadine combination in MCF7 cells collected after 24 hours. **E, F)** Sanguinarine and Withaferin A in MCF7 cells collected after 24 hours. **A, C, E)** Heatmap of excess over CDA is shown with outlier cells bordered in black. **B, D, F)** Comparison of combination estimates and observed viabilities along with goodness-of-fit (GoF) p-value and corresponding optimal Spearman correlation's estimate. Points are colored with the same scale as its corresponding EOCDA matrix. If the GoF p-value > 0.01 for the overall combination, then each point (i.e., dose) is classified as following the dCDA model or not (i.e., likely synergistic or antagonistic behavior). Related to Figure 2

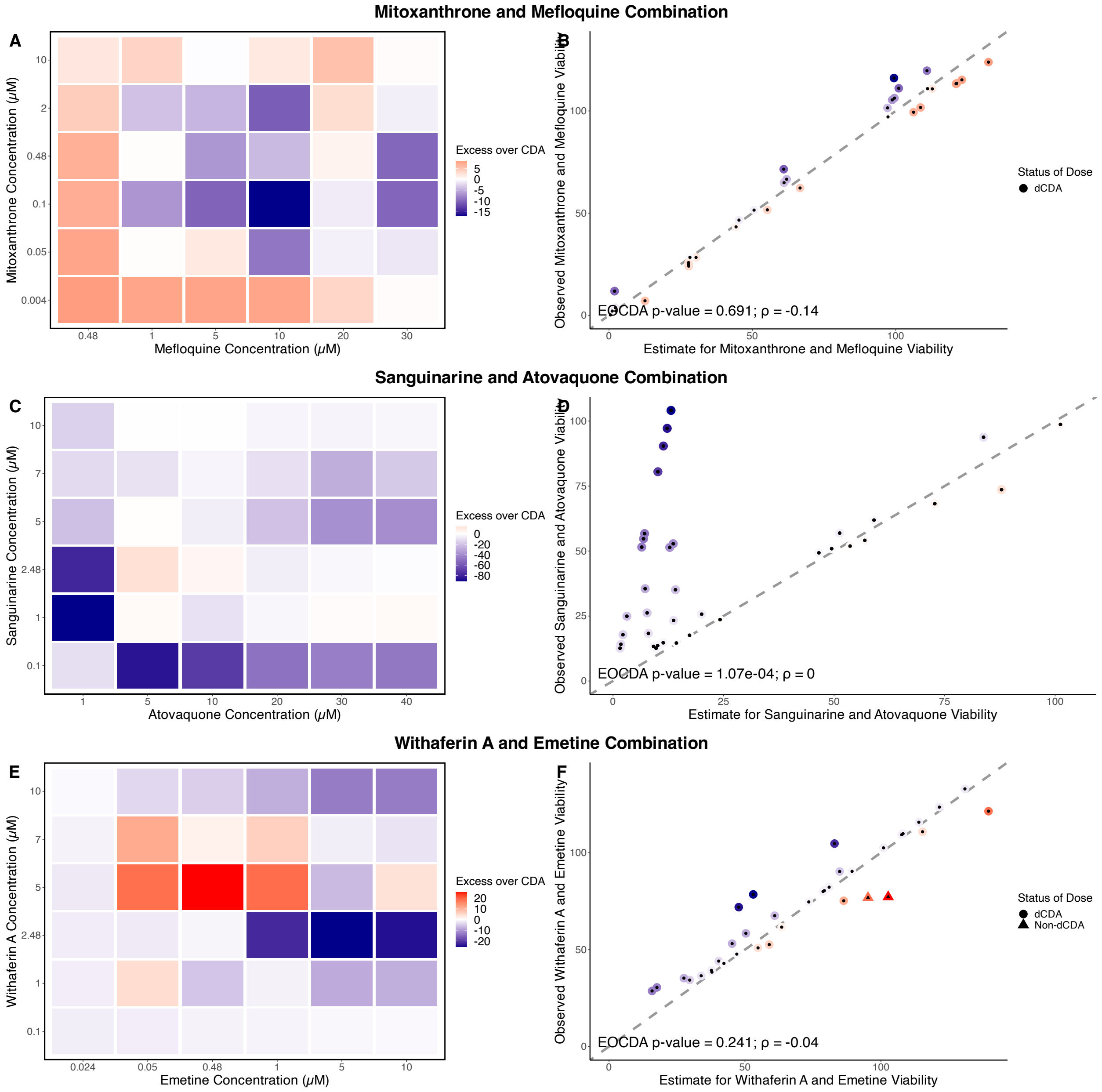

Figure S14: dCDA model results. Each row corresponds to a given combination. **A, B)** Mitoxanthrone and Mefloquine in MCF7 cells. **C, D)** Sanguinarine and Atovaquone combination in MCF7 cells collected after 24 hours. **E, F)** Emetine and Withaferin A in MCF7 cells collected after 24 hours. **A, C, E)** Heatmap of excess over CDA is shown with outlier cells bordered in black. **B, D, F)** Comparison of combination estimates and observed viabilities along with goodness-of-fit (GoF) p-value and corresponding optimal Spearman correlation's estimate. Points are colored with the same scale as its corresponding EOCDA matrix. If the GoF p-value > 0.01 for the overall combination, then each point (i.e., dose) is classified as following the dCDA model or not (i.e., likely synergistic or antagonistic behavior). Related to Figure 2

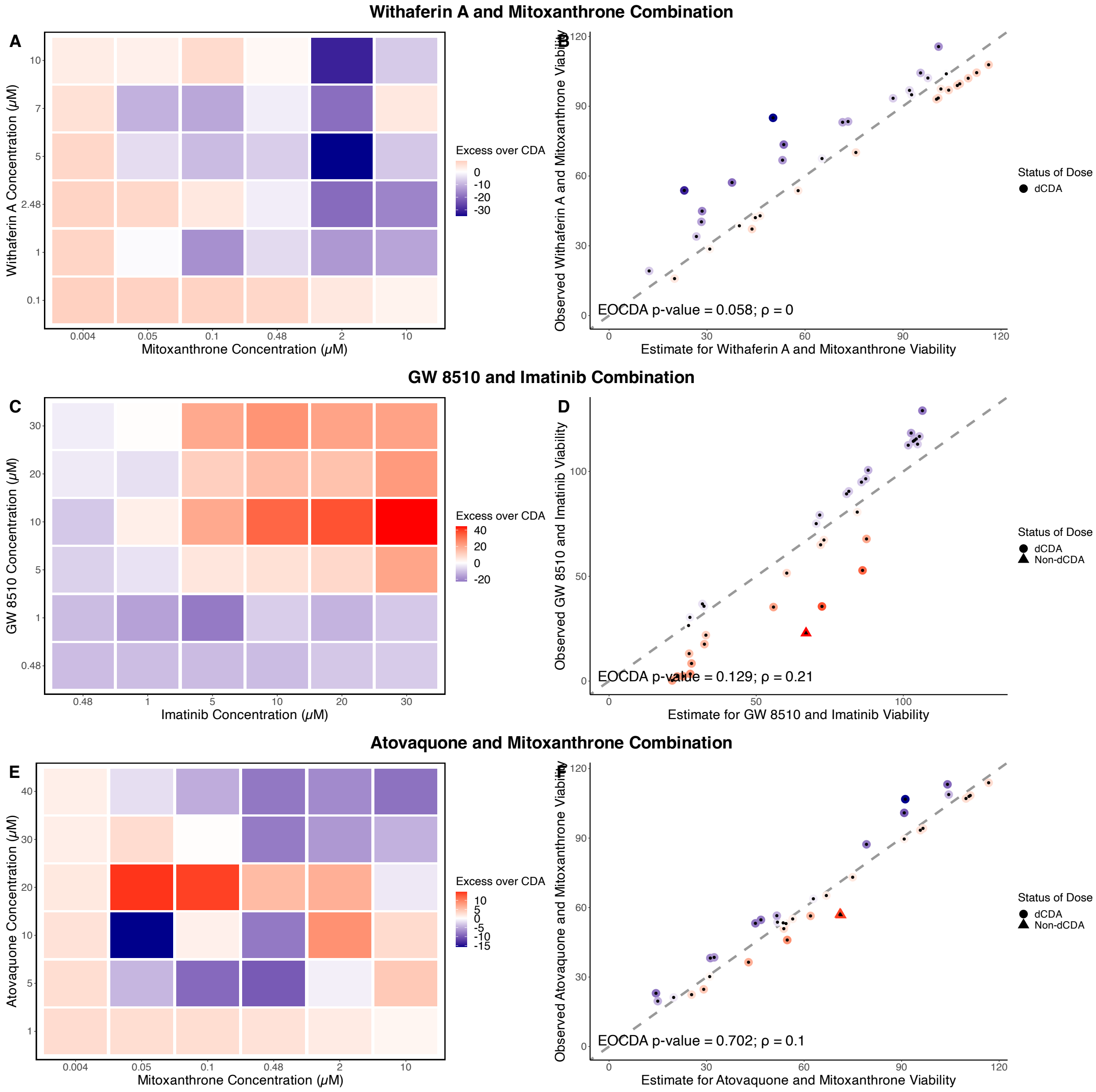

Figure S15: dCDA model results. Each row corresponds to a given combination. **A, B)** Withaferin A and Mitoxanthrone combination in MCF7 cells collected after 24 hours. **C, D)** GW 8510 and Imatinib combination in MCF7 cells collected after 24 hours. **E, F)** Atovaquone and Mitoxanthrone combination in MCF7 cells collected after 24 hours. **A, C, E)** Heatmap of excess over CDA is shown with outlier cells bordered in black. **B, D, F)** Comparison of combination estimates and observed viabilities along with goodness-of-fit (GoF) p-value and corresponding optimal Spearman correlation's estimate. Points are colored with the same scale as its corresponding EOCDA matrix. If the GoF p-value > 0.01 for the overall combination, then each point (i.e., dose) is classified as following the dCDA model or not (i.e., likely synergistic or antagonistic behavior). Related to Figure 2

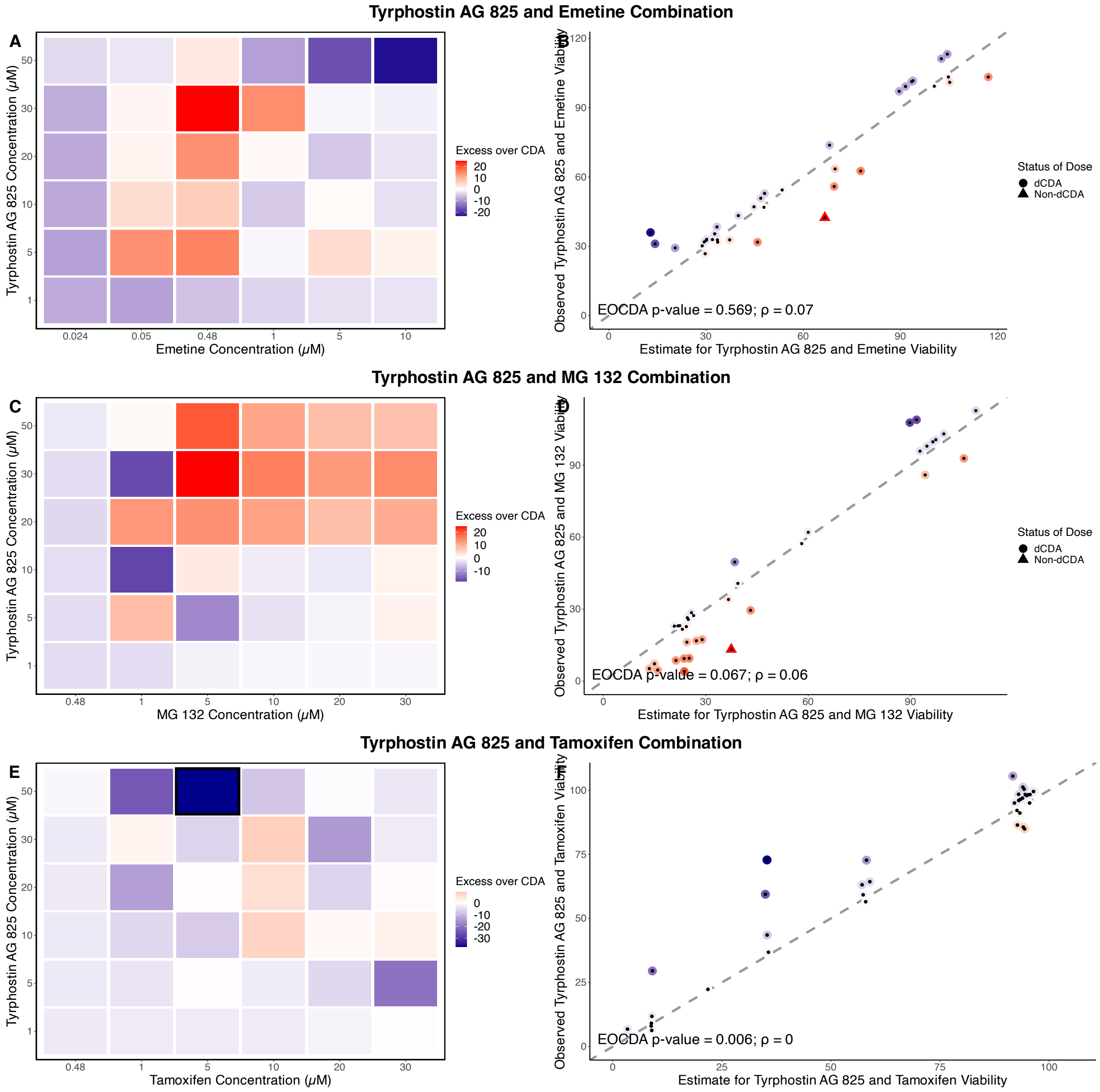

Figure S16: dCDA model results. Each row corresponds to a given combination. **A, B)** Tyrphostin AG 825 and Emetine combination in MCF7 cells collected after 24 hours. **C, D)** Tyrphostin AG 825 and MG 132 in MCF7 cells collected after 24 hours. **E, F)** Tyrphostin AG 825 and Tamoxifen combination in MCF7 cells collected after 24 hours. **A, C, E)** Heatmap of excess over CDA is shown with outlier cells bordered in black. **B, D, F)** Comparison of combination estimates and observed viabilities along with goodness-of-fit (GoF) p-value and corresponding optimal Spearman correlation's estimate. Points are colored with the same scale as its corresponding EOCDA matrix. If the GoF p-value > 0.01 for the overall combination, then each point (i.e., dose) is classified as following the dCDA model or not (i.e., likely synergistic or antagonistic behavior). Related to Figure 2

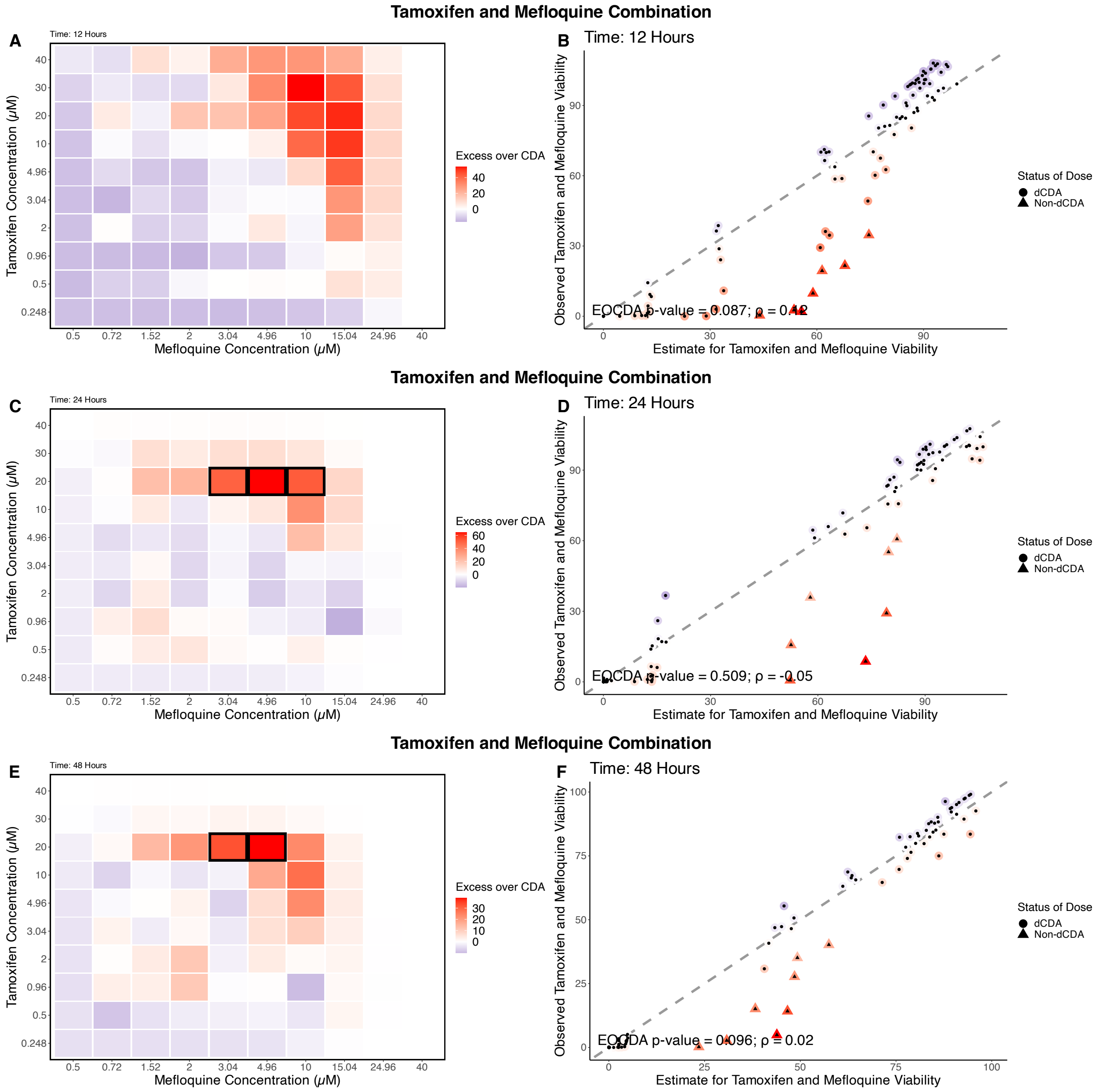

Figure S17: dCDA model results. Each row corresponds to a given combination. **A, B)** Tamoxifen and Mefloquine combination in MCF7 cells collected at 12 hours. **C, D)** Tamoxifen and Mefloquine in MCF7 cells collected at 24 hours. **E, F)** Tamoxifen and Mefloquine in MCF7 cells collected at 48 hours. **A, C, E)** Heatmap of excess over CDA is shown with outlier cells bordered in black. **B, D, F)** Comparison of combination estimates and observed viabilities along with goodness-of-fit (GoF) p-value and corresponding optimal Spearman correlation's estimate. Points are colored with the same scale as its corresponding EOCDA matrix. If the GoF p-value > 0.01 for the overall combination, then each point (i.e., dose) is classified as following the dCDA model or not (i.e., likely synergistic or antagonistic behavior). Related to Figure 2

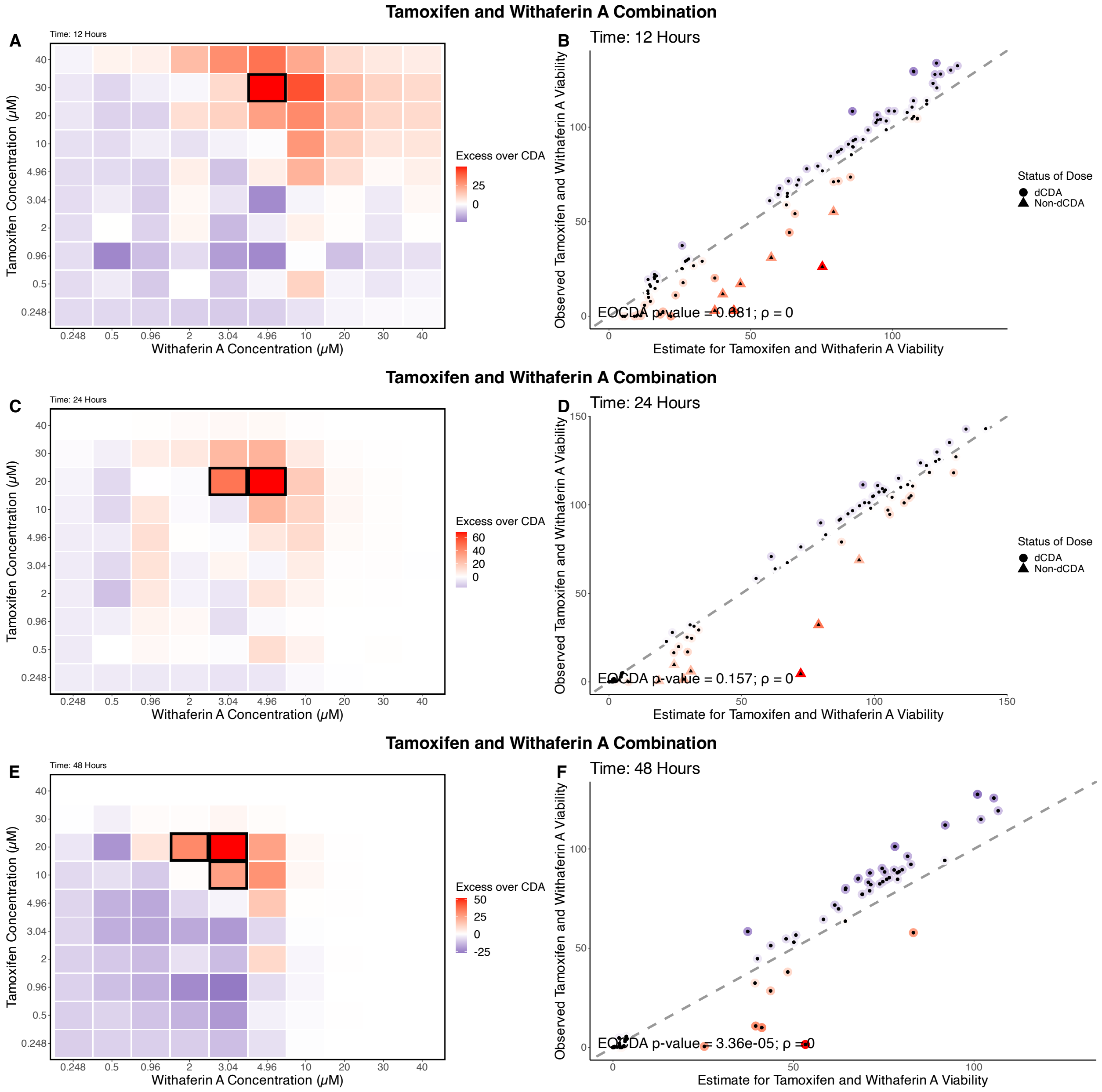

Figure S18: dCDA model results. Each row corresponds to a given combination. **A, B)** Tamoxifen and Withaferin A combination in MCF7 cells collected at 12 hours. **C, D)** Tamoxifen and Withaferin A in MCF7 cells collected at 24 hours. **E, F)** Tamoxifen and Withaferin A in MCF7 cells collected at 48 hours. **A, C, E)** Heatmap of excess over CDA is shown with outlier cells bordered in black. **B, D, F)** Comparison of combination estimates and observed viabilities along with goodness-of-fit (GoF) p-value and corresponding optimal Spearman correlation's estimate. Points are colored with the same scale as its corresponding EOCDA matrix. If the GoF p-value > 0.01 for the overall combination, then each point (i.e., dose) is classified as following the dCDA model or not (i.e., likely synergistic or antagonistic behavior). Related to Figure 2
